# Exclusionary bargaining behavior in 14 countries: Prevalence and predictors

**DOI:** 10.1093/pnasnexus/pgae553

**Published:** 2025-01-28

**Authors:** Andrzej Baranski, Nicholas Haas

**Affiliations:** Division of Social Science and Center for Behavioral Institutional Design, New York University Abu Dhabi, Abu Dhabi 129188, United Arab Emirates; Department of Political Science, Aarhus University, Bartholins Allé 7, Aarhus 8000, Central Denmark Region, Denmark

**Keywords:** bargaining, culture, experiment, gender, ideology

## Abstract

Primates are known to engage in exclusionary behavior, forming alliances to block a minority from accessing scarce resources. Humans are no exception, and examples of exclusionary behavior abound in political, business, and social settings. However, despite its socio-economic relevance, little is known about the prevalence and determinants of such behavior worldwide. Conducting an experimental game in which a group divides resources by majority rule, we document considerable global heterogeneity in exclusionary behavior. Whereas exclusion is modal in some countries, inclusive behavior is the norm in others. Despite significant cross-country variability, we nevertheless find that individual-level characteristics matter similarly across contexts. Men, individuals with a deliberative reasoning style, and ideologically right-leaning individuals, are consistently and substantially more exclusionary. Cross-country differences in the formation of exclusionary alliances correlate with an original Hierarchy Tolerance Index, derived from variables measuring cultural acceptance of power inequalities. Our findings carry important implications for decision-making bodies, as they indicate that the identity of decision-makers and the culture in which they are embedded can affect how equitably resources are divided.

**Significance Statement**

The experience of exclusion from access to resources and opportunities—whether social, political, or economic—can divide individuals and societies and lead to sustained conflict. In order for exclusion to be reduced, however, it is necessary to first measure its prevalence and identify who engages in exclusionary behavior. In this paper, we take a first step by conducting incentivized negotiation experiments across a diverse set of 14 countries around the world. We uncover substantial differences in the likelihood of exclusion across countries. Individual-level characteristics (gender, reasoning style, and ideological orientation) predict who excludes, suggesting that changing who is in power can alter its prevalence. Exploratory analyses suggest that the cultural acceptance of hierarchies and power inequalities correlates positively with exclusionary behavior.

## Introduction

In the midst of scarcity, humans and other primates often engage in exclusionary behavior to secure a share of resources for themselves (1, 2). Excluding others increases the share of resources per member of the beneficiary group, which may confer a survival or reproductive advantage. In human organizations, large and small alike, the formation of alliances and coalitions to advance common interests at the expense of others has therefore long been the subject of theoretical and empirical investigations by scholars in political science (3–5), sociology (6, 7), economics (8, 9), psychology (10, 11), and beyond. For instance, political scientists often aim to understand how political actors (e.g. parties) distribute a scarce resource (e.g. cabinet seats) to advance their joint interests at others’ expense (4, 5, 12); sociologists study “social closure,” or the practice by which members of one group impede others from sharing in available opportunities and resources (7); economists investigate how coalitions form and who they exclude across a number of settings (8, 9, 13–15); and psychologists scrutinize the causes and consequences of ostracism, exclusion, and rejection (10, 11). In this article, we investigate differences in exclusionary behavior across countries, as well as the individual determinants of the decision to exclude others from access to resources.

To this end, we conduct an incentivized laboratory experiment across 14 geographically, culturally, and structurally diverse societies covering all six inhabited continents.^a^ We implement an experimental negotiation game (3) in which participants, who are grouped in triads, distribute a fixed amount of monetary resources. Within a given triad, one participant is randomly selected to distribute the money in any way he or she wishes, as long as a majority of members agree. The process repeats itself until an agreement is reached. Participants are free to share inclusively, but can also form exclusionary alliances if they attract the minimal required support. In our game, there is no incentive to build a reputation for future negotiations. Further, exclusion does not serve the purpose of disciplining members to induce cooperation in a social dilemma (16–19) or expand the surplus to be distributed, and as such, it cannot be justified by appeals to efficiency gains. Thus, we measure exclusion driven by competitive and selfish aims: it harms excluded members, benefits those sharing the resources, and offers no positive externalities, such as enhanced cooperation. While the extant experimental literature (9, 15, 20–31) has substantially advanced our comprehension of social exclusion and its consequences, we still lack an understanding of cross-national differences in exclusion and the individual-level predictors of exclusionary behavior. Investigations of the canonical experimental coalition formation game we study in this paper have been limited almost entirely to the United States or the United Kingdom. These studies do not consider individual-level predictors of exclusion, focusing instead on group behavior. Existing studies are accordingly unable to answer the two critical questions we address in this manuscript. First, does exclusion vary from one society to another? Second, do personal characteristics explain patterns of exclusion?

We document substantial heterogeneity around the world in the willingness to propose exclusionary alliances to share limited resources, as well as in the inequality of resource distributions. In Austria, for example, just under 20% of negotiations result in exclusionary alliances, while these represent 70% of splits in China. Importantly, our results show that previous evidence based almost entirely on the United States and United Kingdom (see meta analysis in Appendix S7) (32, 33) are not representative of behavior globally and indeed overstates willingness to exclude.

Turning to individual-level factors, we expected that individuals’ gender (34, 35), cognitive reasoning style (i.e. deliberative vs. intuitive) (36–38), and ideological orientation (39, 40) would predict their likelihood of engaging in exclusionary behavior. We find strong support for these expectations: men, those who score more highly on a cognitive reflection task (CRT), and politically right-leaning individuals, all exhibit higher levels of exclusion. The stability of these predictors across countries with varying levels of exclusion and in uniform and comparable student samples provides strong evidence for their relevance across contexts. Whereas we interpret gender and ideological orientation effects as stemming from differences in preferences, we view CRT effects as reflecting differences in individuals’ abilities to engage in mental deliberation and think more strategically about the experiment task. In line with our interpretation, gender and ideological orientation remain significant predictors throughout the 15 repetitions of the experiment that participants play, whereas the effect of CRT fades in the last three games—suggesting that subjects learn to reason strategically.

Having uncovered substantial differences across our 14 country sample, a natural question emerges: Can patterns of exclusionary behavior be attributed to cultural differences between countries (41, 42)? Culture is a multifaceted construct (43–45), and the literature offers an abundance of measures. In our exploratory cross-country analysis, we focus on one dimension of culture that we expect will carry particular relevance for exclusionary behavior in our game: the tolerance of hierarchies and power inequalities. Note that in our experiment, the subject who divides resources is exogenously granted decision-making power, meaning that she both is endowed with a higher social ranking and can produce further inequalities through her proposed coalition and division.

We conjecture that subjects in countries that exhibit high cultural acceptance of hierarchy and differences in power will be more likely to engage in exclusionary behavior. To explore our supposition, we constructed an original *Hierarchy Tolerance Index* (HTI), which draws on three different sources and conceptualizations of hierarchy acceptance: Schwartz’s hierarchy value (46), Hofstede’s Power Distance Index (PDI) (43, 47), and a question from the World Values Survey (48) concerning the necessity to obey authority. We find that HTI, and each of its constitutive components, correlates positively with exclusionary behavior in our experimental game: individuals from countries high on HTI are less likely to distribute resources equally and more likely to propose exclusionary alliances. No other cultural variable that we explored revealed such a consistent and high level of correlation.

## The experiments

The workhorse game we implement in the laboratory closely follows a widely studied multilateral negotiation model (3) in which members of a group decide how to divide a common fund. This stylized game is useful for modeling distributive negotiations in which members have opposing interests, meaning that the gain of one person comes at the expense of another. Examples of settings in which opposing interests arise in distributive bargaining are many and far-ranging: for instance, negotiations to distribute cabinet posts in parliamentary democracies, the distribution of budgetary expenditures in legislatures, profit-sharing in business partnerships, climate change reparations for loss and damage, or bankruptcy liquidation negotiations. A common feature across these contexts is that, under majoritarian decision rules, a simple majority may share the benefits while excluding those whose consent is redundant for agreement, but nothing precludes the bargainers from being more inclusive in their distributions.

To implement the majoritarian bargaining game, we follow standard procedures in the literature (9, 22). A comprehensive description of our experimental procedures can be found in Appendix S1. Subjects were placed in groups of three with the objective of dividing a monetary endowment. A member of the group was selected at random to propose a distribution of the endowment, specifying a share for each member. The share offered to each member had to be between zero and the endowment, with the condition that the sum of shares should equal the endowment. Once a proposal was made, the nonproposing members observed the distribution and cast their votes independently. If a proposal received the support of a majority (the proposer is counted as voting in favor), an agreement was reached and negotiations ended. In the event of rejection, the process was repeated—from the random selection of a proposer—until agreement. No group exceeded six negotiation rounds.

Subjects played 15 games in total. In each game, new groups were randomly formed. All interactions were computer-mediated and anonymous, and participants were unaware of the identities of their group members. By keeping identities anonymous and interactions computerized, we ensured that the shares offered by proposing members could not be conditioned on recipients’ observable characteristics. Because all participants have equal probabilities of being the proposer, there is no reason for any one member to be treated differently from another; hence our game is one of ex ante perfect symmetry.^b^ Asymmetries in power arise once a proposer has been randomly selected. Because we randomly rematch participants into new groups for each game and they are anonymous, we ensure that there are no reputational incentives associated with an individual’s behavior.

The experimental instructions were read out loud. Subjects could only proceed with the experiment if they correctly answered two comprehension questions ensuring that they understood that decisions were reached by majority rule and that they were randomly rematched after each game. Our intention was to minimize the possibility of confusion affecting sharing behavior and to ensure that subjects were aware that their actions in one game could not have reputational repercussions in a subsequent one (49). We aimed to keep instructions in different languages as close to each other as possible using the method of back-and-forth translation.

To evaluate sample characteristics and individual-level predictors of exclusionary behavior, we administered a socio-demographic questionnaire, as well as questions on political ideological orientation and a three-item CRT. Our participants (n=1,485) were university students, which is a relatively homogeneous sub-population in terms of socio-economic status, age, and educational level, but which varied substantially in ideology and CRT scores to allow our intended analysis (see Appendix Tables S2 and S3 for details). In addition to the benefits of our data collection strategy enumerated above, we note that interactive experiments like the one conducted in this study require a stable set of participants to be rematched and to play several iterations of the game, which is challenging to conduct outside the confines of a laboratory. Importantly, they require proficiency in the use of computers, which makes college students particularly suitable. Although student samples have been shown to be more selfish and cognitively sophisticated than representative samples, comparative evidence (50) suggests that this does not compromise the external validity of the correlations we aim to study.

This study received approval from the NYU Abu Dhabi Institutional Review Board (no. HRPP-2022-94) and was preregistered on Open Science Framework (see Online Appendix for complete version). Informed consent was obtained from all participants.

## Results

Our analysis aims to capture the prevalence of exclusionary behavior, as well as its drivers, in the sharing of scarce resources. We record a proposed division of resources as *exclusionary* if one—and only one—member of the group is entirely excluded from its proceeds. Such a division captures a scenario in which majority members divide available resources entirely between themselves and allocate nothing to redundant members. It thus reflects a severe—though as we will demonstrate, not entirely uncommon—form of exclusion, where the majority seeks only to maximize its own benefits, which come at the expense of a disempowered minority. Additionally, we measure the related concept of proposal *equality* by calculating its Gini coefficient. To ease interpretation, we rescale the Gini coefficient so that 0 indicates perfect inequality and 100 reflects perfect equality (the formula is provided in Appendix S3). Our analyses are based on all proposals that were made, and also hold when restricting attention to accepted proposals.

### Comparisons across countries

**Fig. 1.**
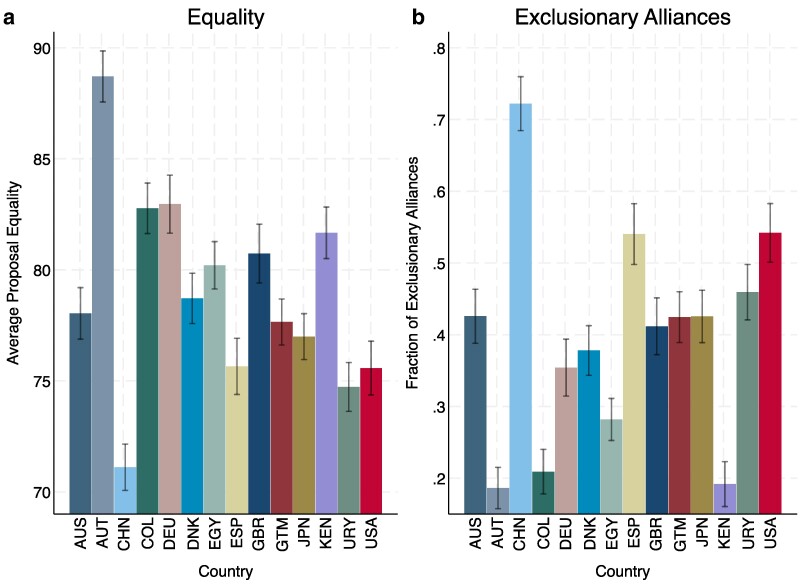
Equality and exclusionary alliances vary across countries. This figure displays average proposal equality a) and the fraction of exclusionary alliances proposed b) by country. We display 95% CI.

We begin with an analysis of patterns in bargaining behavior across the 14 countries in our sample. Figure 1 displays average outcomes across countries, with equality shown in (a) and exclusionary alliances in (b).

A few observations are noteworthy. First, we find a wide degree of variation in both of our outcome measures. The prevalence of exclusionary alliances across countries ranges in proportion from a low of 19% in Austria to a high of 72% in China and with a median of 42%. The gaps are similarly large as regards the average equality of proposals, which ranges from a low of 71 in China to a high of 89 in Austria, with a median value of 78. Kruskal–Wallis tests (shown in Appendix Table S16) indicate that differences across countries are statistically significant for both proposal equality ($\chi^{2}(13)=65.48$, P<0.001) and exclusion ($\chi^{2}(13)=60.82$, P<0.001).

Second, and as is reflected by the above numbers, we observe a strong relationship between proposal equality and exclusion, indicating that both measures indeed capture the same underlying concept of exclusionary alliances. Consistently, pairwise correlations show that the association between the two variables is of substantive and statistical significance: when pooling across the entire sample (r=−0.75, P<0.001), within each country (*r* ranges from −0.65 to −0.84; P<0.001 for all countries), comparing average outcomes within individuals (r=−0.80, P<0.001), or when comparing average outcomes across countries (r=−0.89, P<0.001, see Appendix Table S17).

As detailed in Appendix S7, the vast majority of extant knowledge on majoritarian bargaining comes from two countries: the United States and the United Kingdom. A third observation from our cross-country findings is that our understanding of cross-country differences in exclusionary behavior would be severely limited if we were to only base our conclusions on the three countries (the aforementioned two and Spain) where comparable studies have been conducted. Indeed, extant findings (see Appendix Table S21) from these three countries would expect proposal equality to only range from 71–78 and exclusion from 41%-59%. As detailed above, however, the inclusion of a wider set of countries uncovers far more variation in these outcomes than previously understood. Regression analyses (see Appendix Table S15) on our sample including period fixed effects and clustering at the individual level further indicate that proposal exclusion is higher (P<0.001) and equality lower (P=0.009) in the United States, United Kingdom, and Spain as compared with other countries.

### Comparisons across individuals

We turn our attention to individual-level predictors of exclusionary alliances across countries. We focus on three predictors: an individual’s gender (man or woman, as too few respondents selected “other”), reasoning style as a proxy for the ability to reason strategically, and ideological orientation. We measure the ability to reason strategically using the cognitive reflection task (CRT), as a large number of studies show that it correlates with the ability to identify good strategies that lead to better performance in experimental games (37, 51). We subsequently categorize respondents as exhibiting either “high” CRT (46% of sample: answered two or more of the three questions correctly) or “low” CRT (54%: answered one or fewer of the three questions correctly). In our game, a subject who does not take the time or mental resources to think about the strategic nature of the voting rule may fail to see that an exclusionary alliance can increase her payoffs.

We measure ideological orientation by eliciting responses to four questions on a 1–10 scale. Questions are based on survey items from the European Values Study and World Values Survey (52, 53). They assess views on *economic competition* (1 = competition is good, 1 = competition is damaging), *social welfare* (1 = unemployed should accept any job offer if they wish to retain welfare, 10 = unemployed should be able to refuse any job they do not want), *privatization* (1 = more public companies should be privatized, 10 = more companies should be state-owned), and *redistribution* (reverse coded: 1 = income distribution should be more equal, 10 = there should be more economic incentives for individuals to work harder). We then standardize and center our variables and create an inverse covariance weighted index (54) of ideological orientation, where higher (lower) values correspond to more left- (right-)wing views. Finally, where analyses require a categorical measure of orientation, we classify an individual as “left-leaning” (“right-leaning”) if their support for left-leaning stances was higher (lower) than the median in their country of residence.^c^

**Fig. 2.**
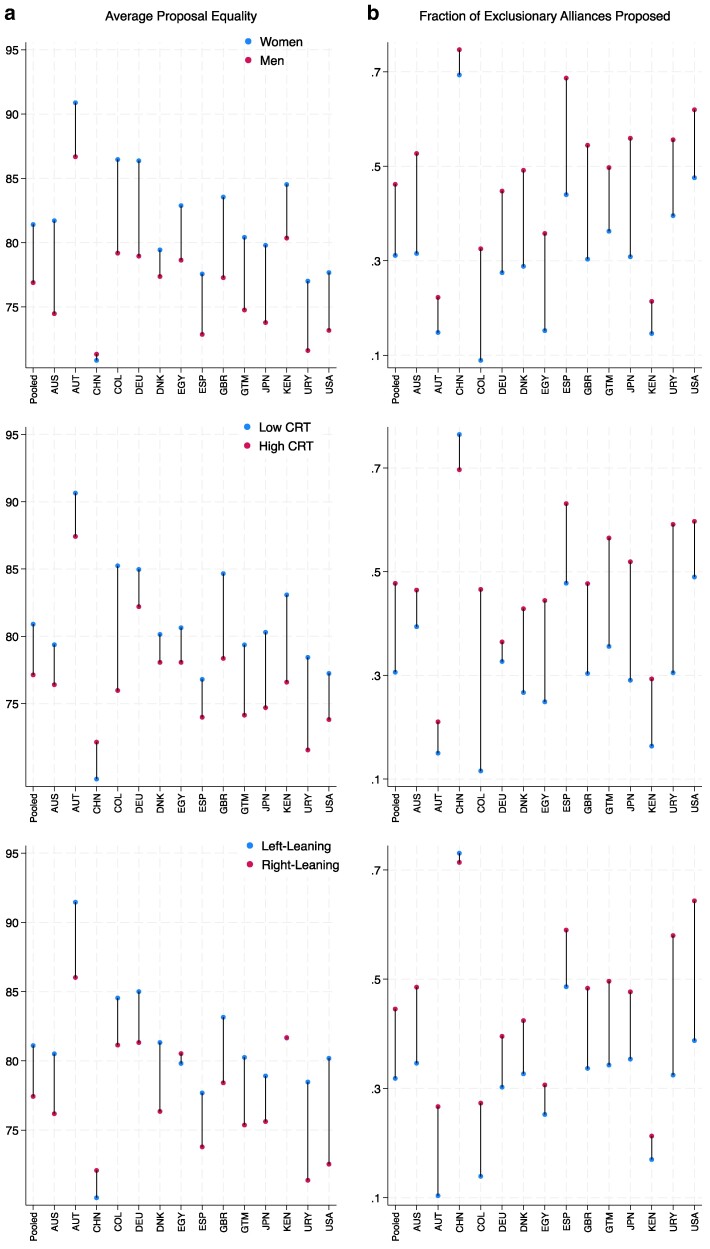
Equality a) and exclusionary alliances b) by gender, CRT, and political attitudes.

Figure 2 plots averages and differences in proposal equality (left panel) and exclusion (right panel) for men and women (top panel), those with low vs. high CRT (middle panel), and those with left- vs. right-leaning ideological orientation (bottom panel). We display results for our pooled sample, as well as for each of the fourteen countries included therein. Results are remarkably consistent across countries: women, individuals with low cognitive reasoning, and those with left-leaning ideological orientations tend to propose more inclusive divisions. Further, we do not observe in any country the opposite effects for either outcome: that is, in no country are women or low CRT or left-leaning individuals statistically *more* likely to propose exclusionary divisions. Table 1 shows that these individual-level predictors are statistically distinguishable from zero and robust to the inclusion of country fixed effects.

**Table 1.** Individual-level predictors of equality and exclusionary alliances.

	(1)	(2)	(3)	(4)	(5)
Panel A. Dependent variable: Equality					
Female	4.51***			3.10***	3.69***
	(0.64)			(0.66)	(0.62)
High CRT score		$-3.77^{***}$		$-3.26^{***}$	$-2.97^{***}$
		(0.64)		(0.64)	(0.64)
Political attitudes index (Right → Left)			3.72***	3.19***	2.36***
			(0.49)	(0.50)	(0.53)
Panel B. Dependent variable: Proposed exclusionary alliance					
Female	$-0.15^{***}$			$-0.10^{***}$	$-0.13^{***}$
	(0.02)			(0.02)	(0.02)
High CRT score		0.17***		0.15***	0.11***
		(0.02)		(0.02)	(0.02)
Political attitudes index (Right → Left)			$-0.09^{***}$	$-0.07^{***}$	$-0.07^{***}$
			(0.01)	(0.01)	(0.01)
*N*	9,121	9,195	9,195	9,121	9,121
Clustering	Indiv	Indiv	Indiv	Indiv	Indiv
FE					Country
Proposals	All	All	All	All	All

*P<0.05, **P<0.01, ***P<0.001. Clustered standard errors in parentheses.

### The role of culture

As detailed in the game-theoretical analysis presented in Appendix S2, any distribution of the surplus can be sustained as an Nash equilibrium in our game. Thus, theory may have little predictive power because bargainers effectively face a coordination problem in deciding which proposals to make that will be acceptable by others (there is no communication in our setting). Hence, in this section we explore the possibility that a shared understanding of cultural values and norms may help subjects coordinate on what a (culturally) acceptable proposal is.

Previous studies on bilateral negotiations have posited that differences in behavior in otherwise identical experimental tasks can be attributed to cultural differences (41, 42). There is no singular measure of culture that could capture the many dimensions on which one society may differ from another. Although significant progress has been made over the last three decades in identifying and quantifying cultural differences between countries, evidence establishing how these measures correlate with negotiation behavior remains scarce and, to the best of our knowledge, altogether absent from the study of multilateral bargaining. In light of our limitations given the size of our sample (N=14), we present an exploratory correlational analysis in this section.

Our first step in this direction is to identify a cultural dimension germane to the task that subjects engage in. We focus on one important characteristic, namely the power asymmetry that arises once a member of the group is selected as the proposer. This can be construed as the formation of a hierarchy within the group, where one person possesses temporary control over resources; others can only accept or reject a proposal, and they have no direct say on how resources should be split. According to Schwartz (46, p,141), “[hierarchy] defines the unequal distribution of power, roles, and resources as legitimate. People are socialized to take the hierarchical distribution of roles for granted.” If asymmetries in power are culturally acceptable, we in turn expect that the exercise of such power in dividing resources will also be perceived as acceptable.

We thus focus on the acceptance of inequalities in power as a relevant dimension on which countries may differ culturally. To measure acceptance of hierarchies and power asymmetries, we construct the *Hierarchy Tolerance Index* (HTI), an original inverse-covariance weighted composite measure that integrates three commonly used sources in cross-cultural research (54): Hofstede’s Power Distance Index (43), Schwartz’s hierarchy dimension (46), and a question from the World Values Survey (48) about obeying authority. We selected these measures due to their widespread use, validation, and relevance for our objective of assessing a country’s tolerance for hierarchy. A detailed description of each variable and the construction of the HTI can be found in Appendix S6.5.

**Fig. 3.**
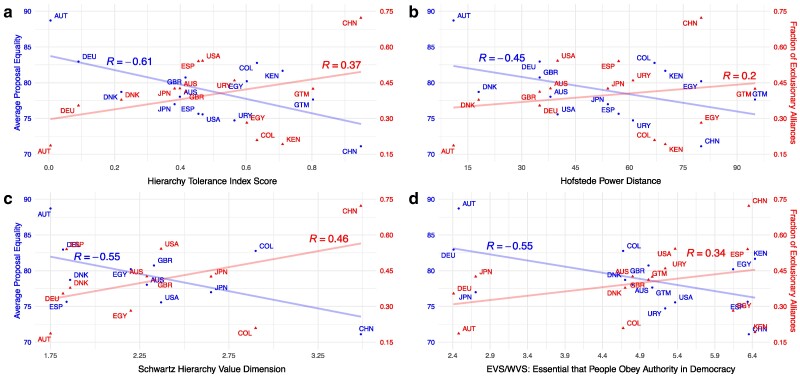
Cultural hierarchy and equality and exclusion in proposals. This figure displays country-level correlations between cultural hierarchy and exclusionary behavior in our experiment. We measure cultural hierarchy using both commonly used measures—Hofstede Power Distance Index b), Schwartz Hierarchy c), and European/World Values Survey data on obeying authority d)—as well as an original inverse-covariance weighted “Hierarchy Tolerance” index based on these measures a). We correlate culture both with average proposal equality (left *y*-axis) and the fraction of exclusionary alliances proposed (right *y*-axis). We also display the Pearson correlation coefficient; Appendix Table S17 reports associated *P*-values.

In Fig. 3a, we plot the HTI against average proposal equality measured on the left *y*-axis and the proportion of exclusionary alliances on the right *y*-axis. In line with our conjecture, we find a positive correlation between exclusion and HTI (R=0.37), and a negative correlation for equality (R=−0.61). A similar pattern holds for each of the three variables comprising the HTI (Fig. 3b–d). The robustness of the correlations is remarkable, especially given that Schwartz’s hierarchy variable is based on teacher and student samples, Hoftstede’s PDI is drawn from responses by employees in a firm, and the WVS is sourced from nationally representative samples.

No other cultural or structural variable that we examined revealed as consistent or high correlations with our bargaining outcomes as compared with those observed for the HTI. Further exploratory analyses on the cultural impact on bargaining, including additional variables, correlation coefficients, and *P*-values, can be found in Appendix Section S6.5. These analyses are consistent with an interpretation of culture as a multidimensional construct, whereby aggregated cultural differences need not explain differences in behavior in specific settings, but contextually relevant components—in our study, tolerance of hierarchies and inequalities—can.

Appendix Section S6.5 also reports results from an investigation into the role of country-level economic inequality, as one may argue that experiencing economic inequality could lead to its acceptance and the mimicking of such behavior in the laboratory. We first note that the HTI and Gini are positively correlated (R=0.75, see Appendix Fig. S4). Surprisingly, however, as shown in Appendix Fig. S6, we find virtually no correlation between a country’s Gini coefficient and the proportion of exclusionary alliances proposed (R=−0.0097), and only a mild negative correlation with proposal equality (R=−0.2). As we discuss at greater length in Appendix Section S6.5, this finding further suggests that HTI might better capture willingness to exclude than alternative structural or cultural variables. In the case of country-level economic inequality, it may be that differences across contexts do not necessarily reflect variation in citizens’ preferences, but rather differences in macro-level factors such as party system, regime type, or historical legacies (55).

### Alternative explanations

In this Section, we detail a few alternative explanations for observed results and explain why we find them unlikely (See Appendix Sections S5 and S6 for all tests and results). First, one potential concern with our results is that the cross-country differences we have documented would disappear as participants learned how to maximize their earnings (32). In the aggregate, we do observe a general trend towards more exclusionary alliances with greater experience. However, the evidence shows that cross-country differences significantly prevail throughout the games as revealed by Kruskal–Wallis tests performed over the course of the 15 games (see Appendix Table S16).

**Fig. 4.**
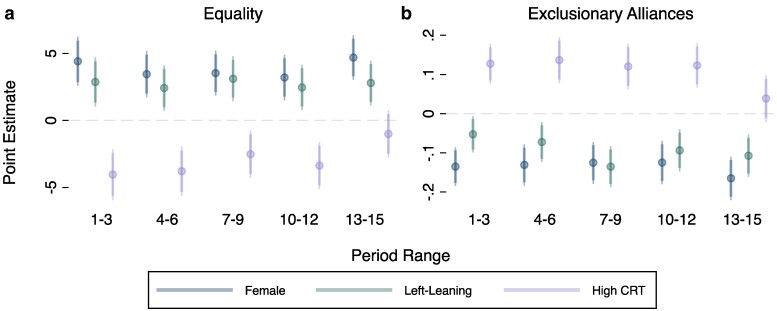
Effects by period range. Coefficients sourced from regressions with all listed variables included, standard errors clustered at the respondent level, and country fixed effects. Results shown for both equality a) and exclusionary alliances b).

Second, we investigate whether the effects of gender, ideological orientation, and reasoning style remain as subjects gain experience. Figure 4 indicates that differences in proposal equality and exclusion based on gender and ideological orientation persist and are statistically distinguishable from zero as the game progresses into later periods of play. The exception is CRT which ceases to predict differences between high and low scorers. As we reflect on at greater length in the subsequent section, this finding supports our interpretation of CRT effects being driven by differences in abilities to reason strategically—which we might expect to become less relevant with experience. Third, as detailed in the previous section and Appendix Section S6.5, we examine a number of alternative cultural and structural correlates of cross-cultural exclusion and find that HTI remains the most robust predictor. Fourth, in Appendix Section S5, we demonstrate that our results are robust to a wide variety of robustness tests and alternative specifications.

## Discussion

Our contribution has been 3-fold. First, we document significant cross-societal diversity in the willingness to propose alliances in which majorities exclude minorities from sharing resources. Previous evidence offered a skewed view, portraying exclusionary allocations as modal, when, indeed, this is not the case in several societies we study. Measurement and documentation of the diversity of human behavior constitutes, in and of itself, a substantial advancement of our knowledge. This is especially important in light of the lack of diversity in human samples that permeates the social sciences (56). Furthermore, if empirical evidence is consistently limited to a subset of developed countries, this can lead to flawed assumptions about behavior in theoretical models, reinforcing a cycle of exclusion in which some members of society are systematically denied representation in scientific studies (57, 58).

Second, we identify three characteristics that correlate systematically with individuals’ predisposition to engage in exclusionary behavior when proposing a division of benefits: gender, ideological orientation, and reasoning styles (deliberative vs. intuitive). Our findings are consistent with women being more responsive and conforming more to social norms of inclusion and egalitarianism than men (35), and plausibly following different moral reasoning processes (34). Regarding ideological orientation, previous research (mainly in the United States) shows that stated norms of fair-sharing vary by ideological orientation, with left-leaning individuals identifying as more egalitarian than right-leaning ones (39, 40). Our study provides evidence that these relationships hold across a wide range of societies; importantly, we do so through an incentivized experiment and thus help bridge the gap between stated preferences and behavior (see Appendix S6.6 for discussion). The stark ideological divide in exclusionary alliances that arises in our setting, which is devoid of context and where the proposer role is assigned by chance and not by merit, is quite intriguing and warrants further investigation to understand its origins. It suggests that a deep-seated tolerance and propensity towards exclusion, regardless of its context, may underpin support for economic and social policies.

Lastly, while the previous two factors highlight the importance of differences in *preferences*, our third factor highlights the importance of *reasoning styles*. In the absence of merit-based justifications for resource distribution, those who are more intuitive reasoners likely started their reasoning process about the game at a focal point of an egalitarian division (59). In contrast, more deliberative and strategic reasoners likely quickly identified exclusionary alliances as a feasible and profit-maximizing alternative and seized on the opportunity. In line with the interpretation that reasoning style affects strategic reasoning, we find that the behavior of high- and low-CRT subjects starts to converge once they have gained enough experience in the game.

Our third contribution is the identification of cultural differences as a plausible reason for the variation between countries in exclusionary bargaining behavior. We find a positive correlation between the proposal of exclusionary alliances in the distribution of resources and the Hierarchy Tolerance Index, an original composite we created consisting of three sources commonly used in cross-cultural analyses. Establishing a link between culture and behavior is a challenging task but one of critical importance in a globalized world where organizational decision-makers often come from different cultural backgrounds. In culturally diverse bodies, views on the sharing of resources can differ dramatically. The cross-country heterogeneity that we uncover opens up several possibilities for future exploration for researchers interested in the role of cultural diversity in human organizations and how culture may interact with personal traits, such as gender.

Our results carry important implications for societies and organizations with collective decision-making bodies. In particular, the gender composition of decision-making bodies has been reported to affect the way resources are used (60, 61) and divided (62). In our experiment, increasing the presence of female decision-makers leads to greater equality and inclusion in the distribution of resources: all-male groups are 45% more likely to form an exclusionary alliance compared to all-female groups. Extrapolating our results to settings outside the laboratory can be challenging because the characteristics and abilities of the men and women that endogenously sort into settings where collective decisions are reached may be different than those of subjects who have been assigned to an experimental task. If, however, the reported gender differences remain after endogenous sorting—and equality in outcomes is an objective that societies pursue—we argue that the case for parity in representation may extend beyond the attainment of equality in access to political participation. It can also foster a more inclusive distribution of public resources.

## Supplementary Material

pgae553_Supplementary_Data

## Data Availability

Data (anonymized) and code required to replicate results presented in this manuscript is available on Harvard Dataverse at the following link: https://doi.org/10.7910/DVN/LJIEMS.

## Notes

a Appendix Table S5 demonstrates the diversity of our sample countries on a number of cultural and structural dimensions. See Appendix Section S6.5 for further discussion and analysis.

b For a game theoretic analysis of game we implement, see Appendix S2.

c See Section Appendix S5.4 for further discussion of this operationalization.

## References

[1] Eisenberg JF, Muckenhirn NA, Rundran R. 1972. The relation between ecology and social structure in primates. Science. 176:863–874. 17829291 10.1126/science.176.4037.863

[2] Goodall J . 1986. Social rejection, exclusion, and shunning among the Gombe chimpanzees. Ethol Sociobiol. 7:227–236.

[3] Baron DP, Ferejohn JA. 1989. Bargaining in legislatures. Am Polit Sci Rev. 83:1181–1206.

[4] Strøm K . 1990. Minority government and majority rule. Cambridge University Press.

[5] Bassi A . 2017. Policy preferences in coalition formation and the stability of minority and surplus governments. J Polit. 79:250–268.

[6] Gamson WA . 1961. A theory of coalition formation. Am Sociol Rev. 26:373–382.

[7] Parkin F . 2018 Strategies of social closure in class formation. In: The social analysis of class structure. Routledge. p. 1–18.

[8] Chatterjee K, Dutta B, Ray D, Sengupta K. 1993. A noncooperative theory of coalitional bargaining. Rev Econ Stud. 60:463–477.

[9] Fréchette G, Kagel JH, Morelli M. 2005. Behavioral identification in coalitional bargaining: an experimental analysis of demand bargaining and alternating offers. Econometrica. 73:1893–1937.

[10] Komorita SS, Chertkoff JM. 1973. A bargaining theory of coalition formation. Psychol Rev. 80:149–162.

[11] Williams KD . 2007. Ostracism. Annu Rev Psychol. 58:425–452. 16968209 10.1146/annurev.psych.58.110405.085641

[12] Diermeier D, Merlo A. 2004. An empirical investigation of coalitional bargaining procedures. J Public Econ. 88:783–797.

[13] Bolton GE, Chatterjee K, McGinn KL. 2003. How communication links influence coalition bargaining: a laboratory investigation. Manage Sci. 49:583–598.

[14] Kamm A, Siegenthaler S. 2022. Commitment timing in coalitional bargaining. Exp Econ. 27:130–154.

[15] Montero M, Sefton M, Zhang P. 2008. Enlargement and the balance of power: an experimental study. Soc Choice Welfare. 30:69–87.

[16] Cinyabuguma M, Page T, Putterman L. 2005. Cooperation under the threat of expulsion in a public goods experiment. J Public Econ. 89:1421–1435.

[17] Maier-Rigaud FP, Martinsson P, Staffiero G. 2010. Ostracism and the provision of a public good: experimental evidence. J Econ Behav Organ. 73:387–395.

[18] Solda A, Villeval MC. 2020. Exclusion and reintegration in a social dilemma. Econ Inq. 58:120–149.

[19] Riedl A, Rohde IM, Strobel M. 2021. Free neighborhood choice boosts socially optimal outcomes in stag-hunt coordination problem. Sci Rep. 11:7745. 33833291 10.1038/s41598-021-87019-y PMC8032720

[20] Diermeier D, Morton R. 2005 Experiments in majoritarian bargaining. In: Social choice and strategic decisions: essays in honor of Jeffrey S. Banks. Springer. p. 201–226.

[21] Fréchette GR, Kagel JH, Morelli M. 2005. Gamson’s law versus non-cooperative bargaining theory. Games Econ Behav. 51:365–390.

[22] Fréchette G, Kagel JH, Morelli M. 2005. Nominal bargaining power, selection protocol, and discounting in legislative bargaining. J Public Econ. 89:1497–1517.

[23] Baranski A, Kagel JH. 2015. Communication in legislative bargaining. J Econ Sci Assoc. 1:59–71.

[24] Agranov M, Tergiman C. 2014. Communication in multilateral bargaining. J Public Econ. 118:75–85.

[25] Miller L, Vanberg C. 2013. Decision costs in legislative bargaining: an experimental analysis. Public Choice. 155:373–394.

[26] Miller L, Vanberg C. 2015. Group size and decision rules in legislative bargaining. Eur J Polit Econ. 37:288–302.

[27] Miller L, Montero M, Vanberg C. 2018. Legislative bargaining with heterogeneous disagreement values: theory and experiments. Games Econ Behav. 107:60–92.

[28] Maaser N, Paetzel F, Traub S. 2019. Power illusion in coalitional bargaining: an experimental analysis. Games Econ Behav. 117:433–450.

[29] Laroze D, Hugh-Jones D, Leininger A. 2020. The impact of group identity on coalition formation. Res Polit. 7:1–7.

[30] Baranski A, Haas N. 2023. The timing of communication and retaliation in bargaining: an experimental study. J Econ Psychol. 96:102621.

[31] Kim DG, Lim W. 2024. Multilateral bargaining over the division of losses. Games Econ Behav. 146:59–76.

[32] Palfrey TR . 2016. Experiments in political economy. In: Kagel JH, Roth AE, editors. The handbook of experimental economics, Vol. 2. Princeton University Press. p. 347–434.

[33] Baranski A, Morton R. 2022. The determinants of multilateral bargaining: a comprehensive analysis of Baron and Ferejohn majoritarian bargaining experiments. Exp Econ. 25:1079–1108.

[34] Gilligan C . 1993. In a different voice: psychological theory and women’s development. Harvard University Press.

[35] Bilén D, Dreber A, Johannesson M. 2021. Are women more generous than men? A meta-analysis. J Econ Sci Assoc. 7:1–18.

[36] Frederick S . 2005. Cognitive reflection and decision making. J Econ Perspect. 19:25–42.

[37] Carpenter J, Graham M, Wolf J. 2013. Cognitive ability and strategic sophistication. Games Econ Behav. 80:115–130.

[38] Brañas-Garza P, Kujal P, Lenkei B. 2019. Cognitive reflection test: whom, how, when. J Behav Exp Econ. 82:101455.

[39] Graham J, Haidt J, Nosek BA. 2009. Liberals and conservatives rely on different sets of moral foundations. J Pers Soc Psychol. 96:1029–1046. 19379034 10.1037/a0015141

[40] Jost JT, Federico CM, Napier JL. 2009. Political ideology: its structure, functions, and elective affinities. Annu Rev Psychol. 60:307–337. 19035826 10.1146/annurev.psych.60.110707.163600

[41] Roth AE, Prasnikar V, Okuno-Fujiwara M, Zamir S. 1991. Bargaining and market behavior in Jerusalem, Ljubljana, Pittsburgh, and Tokyo: an experimental study. Am Econ Rev. 81:1068–1095.

[42] Henrich J . 2000. Does culture matter in economic behavior? Ultimatum game bargaining among the Machiguenga of the Peruvian Amazon. Am Econ Rev. 90:973–979.

[43] Hofstede G . 2001. Culture’s consequences: comparing values, behaviors, institutions and organizations across nations. Sage.

[44] Muthukrishna M, et al 2020. Beyond western, educated, industrial, rich, and democratic (WEIRD) psychology: measuring and mapping scales of cultural and psychological distance. Psychol Sci. 31:678–701. 32437234 10.1177/0956797620916782 PMC7357184

[45] Kaasa A . 2021. Merging Hofstede, Schwartz, and inglehart into a single system. J Cross Cult Psychol. 52:339–353.

[46] Schwartz S . 2006. A theory of cultural value orientations: explication and applications. Comp Sociol. 5:137–182.

[47] Hofstede G, Hofstede G, Minkov M. 2010. Cultures and organizations: software of the mind. 3rd ed. McGraw-Hill Education. http://books.google.de/books?id=o4OqTgV3V00C.

[48] EVS/WVS . 2021. European values study and world values survey: joint EVS/WVS 2017-2021 dataset (joint EVS/WVS).

[49] Smith VL . 2007 Rationality in economics: constructivist and ecological forms. Cambridge University Press.

[50] Snowberg E, Yariv L. 2021. Testing the waters: behavior across participant pools. Am Econ Rev. 111:687–719.

[51] Brañas-Garza P, García-Muñoz T, González RH. 2012. Cognitive effort in the beauty contest game. J Econ Behav Organ. 83:254–260.

[52] ESS . 2017. European values study 2017 master questionnaire. https://europeanvaluesstudy.eu/methodology-data-documentation/survey-2017/full-release-evs2017/documentation-survey-2017/.

[53] WVS . 2017. WVS: 2017-2021 world values survey wave 7 master survey questionnaire. https://www.worldvaluessurvey.org/WVSContents.jsp?CMSID=friends&CMSID=friends.

[54] Anderson ML . 2008. Multiple inference and gender differences in the effects of early intervention: a reevaluation of the abecedarian, perry preschool, and early training projects. J Am Stat Assoc. 103:1481–1495.

[55] Engerman SL, Sokoloff KL. 2006 Colonialism, inequality, and long-run paths of development. In: Understanding poverty. Oxford University Press. 10.1093/0195305191.003.0003.

[56] Henrich J, Heine SJ, Norenzayan A. 2010. The weirdest people in the world? Behav Brain Sci. 33:61–83. 20550733 10.1017/S0140525X0999152X

[57] Bracic A, Callier SL, Price WN. 2022. Exclusion cycles: reinforcing disparities in medicine. Science. 377:1158–1160. 36074837 10.1126/science.abo2788

[58] Mares I, Carnes ME. 2009. Social policy in developing countries. Annu Rev Polit Sci. 12:93–113.

[59] Messick DM . 1993 Equality as a decision heuristic. Cambridge University Press.

[60] Chattopadhyay R, Duflo E. 2004. Women as policy makers: evidence from a randomized policy experiment in India. Econometrica. 72:1409–1443.

[61] Apesteguia J, Azmat G, Iriberri N. 2012. The impact of gender composition on team performance and decision making: evidence from the field. Manage Sci. 58:78–93.

[62] Baranski A, Geraldes D, Kovaliukaite A, Tremewan J. 2024. An experiment on gender representation in majoritarian bargaining. Manage Sci. 70:6622–6636.

