# Supplementary material for: Exclusionary bargaining behavior in 14 countries: Prevalence and predictors
Source: PNAS Nexus. 2025 Jan 28;4(1):pgae553. doi: 10.1093/pnasnexus/pgae553 (PMC11773605; doi:10.1093/pnasnexus/pgae553)
Supplement: pgae553_Supplementary_Data [file pgae553_supplementary_data.pdf]

Supplementary Information for *Exclusionary Bargaining Behavior in 14 Countries: Prevalence and Predictors*

Table of Contents

S1 Experimental Procedures **A3**
S1.1 Subject Recruitment A3
S1.2 Session Protocol A3
S1.3 Experimental Software A3
S1.4 Subject Payments A3
S1.5 Language A4
S1.6 Instructions (English, UK Sample) A5

S2 Game Theoretic Model and Equilibrium Notions **A7**

S3 Calculating the Gini coefficient and Equality Measure **A7**

S4 Summary Statistics **A9**

S5 Robustness **A11**
S5.1 Robustness to Different Specifications A11
S5.2 Robustness to Different Weighting Exercises A11
S5.3 Robustness to Different CRT Variable Operationalization A11
S5.4 Robustness and Discussion of Ideological Orientation Operationalization A13

S6 Additional Results **A15**
S6.1 Country-level Results A15
S6.2 Group Composition A15
S6.3 Comparison of Results by Whether Previous Study Conducted in Country A15
S6.4 Learning and Over-Time Changes A18
S6.5 Exploring Cultural and Structural Correlations A22
S6.6 Stated and Revealed Preferences A32

S7 Mini Meta-Analysis **A34**

S8 Deviations from Pre-Analysis Plan **A36**

S9 Pre-Analyis Plan **A37**
S9.1 Study Information A37
S9.2 Design Plan A38
S9.3 Sampling Plan A38
S9.4 Variables A40

S9.5 Analysis Plan A44

S10 Acknowledgements **A46**

S1 Experimental Procedures

In this section, we describe the subject recruitment protocol, how the sessions were conducted, the experimental software used, and subject compensation. We also provide a sample of the instructions in English. This study received NYUAD Institutional Review Board approval (HRPP-2022-94).

S1.1 Subject Recruitment

In every laboratory, we recruited subjects who were part of the laboratory participant pool. Invitation emails did not include any details about the content of the experiment; they only advertised an expected duration of 1.5 hours and the respective fixed participation fees. The recruitment process was managed with Orsee[1] and hRoot[2] when available. Subjects participated only in one session.

S1.2 Session Protocol

Subjects entered the laboratory and were assigned to computer terminals. Communication and the use of cellphones were strictly prohibited. In each session, the following steps took place:

1. Distribution and collection of participation consent forms;
2. Reading out loud of experimental instructions;
3. Two question comprehension quiz. The software allowed subjects to proceed only after they had answered correctly;
4. Subjects were allowed to ask questions in private to the experimenter;
5. 15 repetitions of the bargaining game. New groups are formed at random. Within a game, subjects are assigned ID numbers 1,2, and 3 so that they know who made a proposal and how each voted. When a new group is formed, the ID numbers are randomly assigned and new groups are randomly formed. It is impossible for subjects to identify each other across games.
6. Sociodemographic questionnaire;
7. Payments in private.

The sessions typically lasted between 50 and 70 minutes.

S1.3 Experimental Software

All interactions were computerized. The experiment was programmed in zTree[3] and oTree[4].

S1.4 Subject Payments

Subjects were paid a show-up fee (fixed) and for the share they received in 2 of the 15 periods that they played. Payment periods were randomly selected at the end of each experimental session.

In determining subject payments in each sample, we faced several constraints. First, laboratories differ in their fixed participation fee requirements and the average payment requirements. Compensation levels are set to reflect fair compensation practices and attract enough participants.

We adhered to the policies of each laboratory in this regard. In doing so, we cannot fully control incentives to reflect differences in purchasing power between countries. However, our subjects are of similar socioeconomic status within their countries, and the payment schemes in each lab are aimed at their own population, to attract participants and compensate them *fairly.* Therefore, we believe that by following laboratory guidelines, we control for possible incentive differences better than if we used purchasing power parity conversions.

Below, we provide a table with the mean earnings by country, the purchasing power parity (PPP) conversion factor for 2022 (the U.S. is the base level, i.e. PPP=1), and the mean earnings accounting for purchasing power differences.

Table S1: **Mean Earnings and Purchasing Power**

Country	Mean Earnings	PPP Conversion Factor	PPP-adjusted Earnings
Australia	30	1.4	21.9
Austria	26	0.7	37.3
China	90	3.8	23.7
Colombia	35,000	1345.7	26.0
Denmark	160	6.2	26.0
Egypt	230	4.0	57.8
Germany	26	0.7	37.4
Guatemala	140	3.2	43.9
Japan	4,000	94.9	42.1
Kenya	900	41.9	21.5
Spain	26	0.6	44.9
United Kingdom	14	0.7	21.5
United States	28	1.0	28.0
Uruguay	750	25.8	29.1

Notes: Mean earnings are displayed in the lcoal currency. The PPP Conversion Factor is obtained from the World Development Indicators database [5] for the year 2022. The PPP Conversion Factor base level is the United States.

S1.5 Language

The experimental instructions and software were written in the language of instruction of the university in which the experiment was carried out. The English instructions were translated into Chinese, Spanish, Japanese, and German, using back and forth translation to ensure consistency.

S1.6 Instructions (English, UK Sample)

Experiment Instructions

This is an experiment in the economics of decision making. We follow a no-deception ethical policy; hence these instructions fully describe the experiment. Before participating please also read the consent form and sign with your signature and the date as well as your name in block capitals.

A Brief Overview of the Experiment

In this experiment you will be part of a group of 3 people. One of you will be asked to propose a distribution of 30 tokens (equivalent to 15 GBP) among the members of your group. Proposals are voted up or down according to the simple majority rule. In case the current proposal is rejected, the members of the same group proceed to another proposal and voting round until one allocation is approved. The details of the experiment follow.

The Details of the Experiment

As expressed above, this experiment involves two main components **(1) proposal, and (2) vote.** We proceed to fully explain each of them.

(1) Proposal

The computer will randomly choose one of you to be the proposer of a distribution of 30 token. In this stage the proposer submits a division of the 30 tokens.

(2) Voting

You will observe how much the proposer assigned to each member of the group. You can then click "accept" or "reject". For approval, the proposal requires a simple majority (at least 2 votes). The proposer will automatically be counted as a voting in favor.

If rejected: every member in your group will proceed to stage (2) with a member randomly selected as proposer. Feedback on the previous proposal, the voting result, and who was the proposer will be given to you.

The process repeats itself until an allocation of the total fund is approved.

If approved: the result will be binding. Next, you will then be matched into new groups to repeat the stages (1)-(2). You will participate in a total of 15 periods. In each period, you will be randomly reassigned into a group of 3 people, with your subject number for each period determined randomly as well. Thus, while your subject number will remain the same for all rounds *within* a given period, it will change across periods: in period 1 you can be subject 3, and in period 2 you can be subject 1.

Your Earnings

Only 2 of the 15 periods will be randomly selected to count for payment. Your earnings (E) are then given by the shares you received in those periods plus the show up fee of £5. Each token earned is converted into 50 pence.

Example:

Below, we provide an example for you to understand how the payoffs of the experiment work.

Consider a 3-person group with 30 tokens to divide. The proposer allocates 10 tokens to subject 1, 7 tokens to herself, and 13 tokens to subject 2. If Subject 1 votes in favor and Subject 2 against (the proposer is automatically counted in favor), then the proposal is approved. The payments subjects would receive if this period was selected for payment are the offered shares in the approved proposal. Note however that votes could have been different in which case a new round would take place.

Review of the experiment

1. Everyone is randomly assigned into groups of 3
2. There are 30 tokens to divide. Each token is equivalent to 50 pence.
3. One of you will be randomly chosen as the proposer.
4. Once a proposal is made, voting will take place.
5. If a majority accepts, the allocation is binding, and you will wait in standby until the other groups in your session decide on an allocation.
6. If a majority rejects, the process repeats itself until a given allocation is accepted.
7. Once an allocation is accepted, you will start a new period with randomly selected members. 2 of the 15 periods of play will be chosen randomly for payment.

What should you do? If we knew the answer to this question, we would not need to run an experiment.

After the Experiment

There will be a brief survey where we ask for you some follow up questions. After finishing the survey a final screen will appear with your earnings displayed at the lower end of the screen

S2 Game Theoretic Model and Equilibrium Notions

The game we study in the laboratory has been analyzed under the lense of game theory in a multitude of studies[6–8]. In this section, which is relevant to those interested in formal modeling, we present three well-known equilibrium results under the parameters we have chosen (i.e., 3 players, majority rule, no shrinking of the pie with agreement delay). For the theoretical characterization of equilibrium, we assume that players are risk neutral and that their utility function is given by $u(x) = x$ (where x is money). Perpetual disagreement results in 0 earnings. We denote by F the total money to be divided. Let (s_1, s_2, s_3) represent a distribution of the total fund ($s_i \geq 0$) where $s_1 + s_2 + s_3 = F$.

Proposition 1. *1. Any distribution (s_1, s_2, s_3) is a Nash equilibrium outcome of the game.*

2. Any distribution (s_1, s_2, s_3) is a subgame perfect Nash equilibrium outcome of the game.

Proof. To see that any (s_1, s_2, s_3) is a Nash equilibrium, all we need to show is that there is a profile of strategies that lead to outcomes (s_1, s_2, s_3) such that no player can deviate profitably. Consider the following strategies: proposers propose (s_1^*, s_2^*, s_3^*) and all three players vote in favor, and vote against any other proposal. It is clear that the proposer has no incentive to propose any other distribution, since it will be rejected perpetually, leading to a payoff of 0. Because all members vote in favor of (s_1^*, s_2^*, s_3^*), no voter can individually deviate and change the outcome. Hence, we have shown that there are no profitable deviations from this strategy, and because (s_1^*, s_2^*, s_3^*) is any arbitrary distribution, the result follows.

For the proof of why any distribution of the fund is also a subgame perfect Nash equilibrium , the interested reader may consult[9]. The proof relies on constructing off-equilibrium punishment strategies akin to those in repeated games.

Finally, we turn to the notion of stationary subgame perfect equilibrium (SSPE). Stationarity requires players to enact identical strategies in every round, meaning they cannot condition their strategies on the history of play. The following proposition is taken from[6]:

Proposition 2. *In any stationary subgame perfect Nash equilbrium the following hold*

1. *The proposer offers $F/3$ to one other member with equal probability.*
2. *A voter who receives $F/3$ or more votes in favor.*
3. *The proposer keeps $2F/3$.*

For a proof, see[6] or[7]. As can be seen, the outcome of the game is an exclusionary alliance under the SSPE. The proposer keeps 2/3 of the fund, a share twice as large as that of the member included in the coalition.

S3 Calculating the Gini coefficient and Equality Measure

In this section we explain how we derive our measure of equality. Let E denote equality, and G denote the Gini coefficient. We calculate equality as $E = 100(1 - G)$.

The Gini coefficient is calculated in the standard way[10] by taking the area between the Lorenz curve and the line of perfect equality (shaded region in Figure S1 labeled A), and dividing it by 1/2. Consider the specific case of bargaining to divide a monetary amount (normalized to 1) between a

group of three members, as shown in Figure S1. Let s_1 denote the lowest share, s_2 the median share, and s_3 the highest share. The Lorenz curve plots the cumulative proportion of shares received by the cumulative proportion of players in the group, ordered from the recipient of the lowest share to the highest share.

Note that in order to obtain the area below the line of perfect equality (45° line) and the Lorenz curve, it is equivalent to take the area above the y-axis and below the Lorenz curve and subtract it from 1/2. It is easy to see that the area below the Lorenz curve can be decomposed into the area of three right triangles and two rectangles.

After simplifying, we obtain the area below the Lorenz curve to be $A = s_1/6(4s_1 + 2s_2)$. The area between the Lorenz curve and the line of equality is $1/2 - A$. Thus, we have that

$$G = 1 - 2A\,,$$

which we can substitute into the formula for equality to obtain

$$E = 200A\,.$$

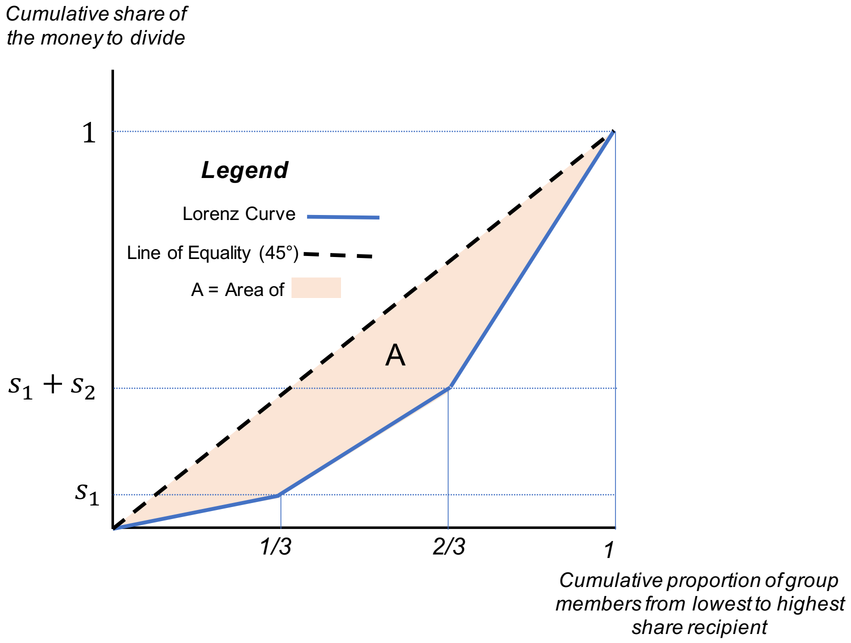

Figure S1: **Lorenz Curve Example for the Computation of the Gini Coefficient**

S4 Summary Statistics

Table S2: **Number of Participants, Periods, and Sessions, Overall and by Country**

Country	# Participants	# Sessions	# Sub-Sessions	# Periods	# Rounds	# Groups
Australia	99	5	10	75	173	495
Austria	114	5	10	75	166	570
China	96	4	8	60	109	480
Colombia	105	5	12	75	158	525
Denmark	114	10	10	150	285	570
Egypt	141	11	12	165	325	705
Germany	90	7	7	105	184	450
Guatemala	123	7	13	105	207	614
Japan	120	6	12	90	165	600
Kenya	102	7	7	105	178	510
Spain	90	2	10	30	70	450
United Kingdom	99	4	11	60	123	495
United States	93	5	9	75	142	465
Uruguay	99	7	9	105	211	495
**Overall**	**1,485**	**85**	**140**	**1,275**	**2,496**	**7,424**

Notes: Sub-sessions are matching groups within a session in which subjects are sampled to be rematched with each other. Sessions and sub-sessions were always composed of 9 subjects or more.

Table S3: **Summary Statistics by Country (Demographics)**

	**Country**													
	AUS	AUT	CHN	COL	DNK	EGY	DEU	GTM	JPN	KEN	ESP	GBR	USA	URY
	(1)	(2)	(3)	(4)	(5)	(6)	(7)	(8)	(9)	(10)	(11)	(12)	(13)	(14)
Age	25.7	24.4	23.1	21.9	23.8	21.5	23.3	20.3	20.4	24.3	21.2	22.1	20.7	20.0
Fraction Women	0.6	0.5	0.5	0.5	0.5	0.4	0.5	0.5	0.5	0.3	0.6	0.6	0.5	0.6
Fraction Who Volunteer	0.5	0.3	0.6	0.3	0.5	0.6	0.4	0.3	0.3	0.7	0.3	0.4	0.7	0.4
Unemployed Can Refuse Job (1-10)	5.1	6.0	6.1	6.9	5.3	4.7	5.2	6.8	5.4	3.4	5.6	4.7	5.2	5.2
Competition is Damaging (1-10)	3.8	4.6	3.8	3.8	3.8	3.7	4.0	1.9	3.9	3.0	4.1	3.8	3.3	2.8
More Economic Inequality (1-10)	5.5	3.6	5.9	6.2	4.8	6.4	4.2	8.8	6.2	7.3	5.4	5.4	5.1	8.0
More Companies State-Owned (1-10)	5.1	6.4	5.3	5.6	5.0	5.0	5.8	2.5	4.4	4.9	5.7	5.4	4.9	3.7
Fraction High CRT	0.4	0.6	0.6	0.3	0.7	0.2	0.7	0.3	0.6	0.2	0.4	0.6	0.5	0.5
Observations	99	114	96	105	114	141	90	123	120	102	90	99	93	99

Table S4: **Average Proposer Outcomes by Country**

	**Country**													
	AUS	AUT	CHN	COL	DNK	EGY	DEU	GTM	JPN	KEN	ESP	GBR	USA	URY
	(1)	(2)	(3)	(4)	(5)	(6)	(7)	(8)	(9)	(10)	(11)	(12)	(13)	(14)
Fraction MWC	0.43	0.19	0.72	0.21	0.38	0.28	0.35	0.42	0.43	0.19	0.54	0.41	0.54	0.46
Average Equality	78.04	88.71	71.11	82.77	78.71	80.20	82.96	77.65	76.99	81.67	75.65	80.73	75.58	74.73

Table S5: **Summary Statistics by Country (Cultural and Structural Measures)**

	AUS	AUT	CHN	COL	DEU	DNK	EGY	ESP	GBR	GTM	JPN	KEN	URY	USA
Hierarchy Index	0.4	0.01	0.95	0.63	0.09	0.22	0.6	0.45	0.42	0.8	0.38	0.71	0.56	0.47
Hofstede PDI	0.32	0	0.82	0.67	0.29	0.08	0.82	0.55	0.29	1	0.51	0.7	0.6	0.35
Schwartz Hierarchy	0.31	0	1	0.66	0.04	0.06	0.26	0.05	0.33		0.52			0.36
WVS/EVS Obey	0.59	0.02	0.98	0.56	0	0.57	0.93	0.98	0.65	0.66	0.07	1	0.7	0.74
Parking Ticket	0	1.2	2.4	0	0.68	0	5	2.6	0.04	0.11	0	2.2	1.7	
Blood Donation	39		8.6	13	57	53	9.2	36	35	0.36	29	5.3	16	35
Western Church	7.1	7.4	0	1.9	7.3	5.5	0	4.1	8.5	0.91	0	0	6.2	5.4
Kinship Intensity	-1	-1	0.16	-1.4	-1	-1.3	1.4	-1.5	-1.3	-0.64	-0.93	0.59	-1.6	-1.2
Cousin Marriage	-0.46		1.1	0.72	-0.8		3.4	1.4	-0.92		1.6		0.96	-1.6
Group Trust	1.2		-1.3	-0.17	0.35		-1.9	0.05	1.2		-0.54		0.45	1.5
Hofstede Individualism	0.99	0.58	0.16	0.08	0.72	0.8	0.22	0.53	0.98	0	0.47	0.22	0.35	1
Country Gini	35	30	42	51	31	28	32	36	34	49	32	48	42	41
GDP Per Capita	50817	49371	16676	14609	49815	49364	12983	38239	44001	8133	42860	3517	22272	59609
Gender Gap	0.97	0.98	0.92	0.98	0.98	0.97	0.97	0.97	0.97	0.98	0.98	0.97	0.98	0.98
Rule of Law	1.8	1.8	-0.22	-0.32	1.6	1.9	-0.41	0.98	1.6	-1	1.4	-0.53	0.63	1.7
WEIRD Country	1	1	0	0	1	1	0	1	1	0	0	0	0	1
PPP	22	37	24	26	37	26	58	45	21	44	42	21	29	28

Notes: This table presents country-level cultural measures (see Appendix Section S6.5 for more details, including information on sources). Because these variables are exclusively used to explore correlations, and to enhance comparability with other research, where possible data sourced from extant work remains on their original scales. Variables used in the construction of our Hierarchy Tolerance Index (PDI, Schwartz, and WVS/EVS Obey) were normalized. PPP refers to Purchasing Power Parity Adjusted Mean Earnings.

S5 Robustness

S5.1 Robustness to Different Specifications

Table S6 probes the robustness of our pooled sample findings to a number of different specifications. We observe that results are robust to limiting analyses to only accepted proposals (column 1), including country and period fixed effects (column 2), limiting analyses to only the final five (column 3) or final (column 4)[4] period(s) of play, to clustering by session (column 5) and matching group (column 6), to controlling for the ratio between the show-up fee and total fund amount being negotiated in a country (column 7), to controlling for Purchasing Power Parity Adjusted Mean Earnings (column 8), and to session fixed effects (column 9).

Table S6: **Equality, Exclusionary Alliances, and Individual-Level Predictors (Robustness)**

	(1)	(2)	(3)	(4)	(5)	(6)	(7)	(8)	(9)
**Panel A. Dependent Variable: Equality**									
Female	3.43***	3.78***	4.12***	5.14***	3.69***	3.69***	3.69***	3.69***	3.88***
	(0.62)	(0.62)	(0.77)	(1.31)	(0.57)	(0.57)	(0.62)	(0.62)	(0.55)
High CRT Score	-2.78***	-2.93***	-2.03**	-0.80	-2.97***	-2.97***	-2.97***	-2.97***	-2.99***
	(0.64)	(0.63)	(0.78)	(1.25)	(0.67)	(0.69)	(0.64)	(0.64)	(0.69)
Political Attitudes Index									
(Right → Left)	2.54***	2.34***	2.47***	2.48*	2.36***	2.36***	2.36***	2.36***	2.43***
	(0.50)	(0.53)	(0.62)	(1.18)	(0.46)	(0.49)	(0.53)	(0.53)	(0.45)
**Panel B. Dependent Variable: Proposed Exclusionary Alliance**									
Female	-0.12***	-0.13***	-0.15***	-0.19***	-0.13***	-0.13***	-0.13***	-0.13***	-0.12***
	(0.02)	(0.02)	(0.03)	(0.04)	(0.02)	(0.02)	(0.02)	(0.02)	(0.02)
High CRT Score	0.10***	0.11***	0.08**	0.02	0.11***	0.11***	0.11***	0.11***	0.11***
	(0.02)	(0.02)	(0.03)	(0.04)	(0.02)	(0.02)	(0.02)	(0.02)	(0.02)
Political Attitudes Index									
(Right → Left)	-0.07***	-0.07***	-0.08***	-0.07*	-0.07***	-0.07***	-0.07***	-0.07***	-0.08***
	(0.02)	(0.01)	(0.02)	(0.03)	(0.01)	(0.01)	(0.01)	(0.01)	(0.01)
N	7,363	9,121	3,097	627	9,121	9,121	9,121	9,121	9,121
Clustering	Indiv	Indiv	Indiv	Indiv	Session	Matching	Indiv	Indiv	Session
FE	Country	Country, Period	Country	Country	Country	Country	Country	Country	Session
Proposals	Accepted Only	All	Periods 11-15	Period 15 Only	All	All	All	All	All
Controls							Show-up/fund	PPP	

Notes: *$p < 0.05$, **$p < 0.01$, ***$p < 0.001$. Standard errors clustered at the specified level in parentheses. PPP refers to Purchasing Power Parity Adjusted Mean Earnings.

S5.2 Robustness to Different Weighting Exercises

Tables S7, S8, and Table S9 further demonstrate the robustness of our findings to additional specifications and weighting exercises. They probe robustness to weighting each country (Table S7) and proposer (Table S8) in our sample equally, and to only counting each proposer once in our sample (Table S9, done by taking as dependent variable the average proposal equality and exclusion for a given individual in all periods when they were the proposer).

S5.3 Robustness to Different CRT Variable Operationalization

Table S10 replicates CRT results using a categorical, as opposed to a dichotomous, operationalization of cognitive reasoning based on CRT score. We find that proposal equality decreases, and

[4]As noted in the main text, CRT score is not a statistically significant predictor in later periods of play.

Table S7: **Equality, Exclusionary Alliances, and Individual-Level Predictors (with Country Weighting)**

	(1)	(2)	(3)	(4)	(5)	(6)	(7)	(8)
**Panel A. Dependent Variable: Equality**								
Female	4.52***			3.09***	3.67***	3.36***	3.73***	4.11***
	(0.64)			(0.67)	(0.62)	(0.63)	(0.62)	(0.76)
High CRT Score		-3.74***		-3.23***	-2.93***	-2.69***	-2.89***	-2.09**
		(0.64)		(0.64)	(0.64)	(0.65)	(0.64)	(0.78)
Political Attitudes Index								
(Right → Left)			3.65***	3.12***	2.39***	2.56***	2.38***	2.44***
			(0.48)	(0.49)	(0.51)	(0.51)	(0.51)	(0.62)
**Panel B. Dependent Variable: Proposed Exclusionary Alliance**								
Female	-0.15***			-0.10***	-0.13***	-0.12***	-0.13***	-0.15***
	(0.02)			(0.02)	(0.02)	(0.02)	(0.02)	(0.03)
High CRT Score		0.17***		0.15***	0.11***	0.10***	0.11***	0.08**
		(0.02)		(0.02)	(0.02)	(0.02)	(0.02)	(0.03)
Political Attitudes Index								
(Right → Left)			-0.09***	-0.07***	-0.07***	-0.07***	-0.07***	-0.08***
			(0.01)	(0.01)	(0.02)	(0.02)	(0.01)	(0.02)
N	9,121	9,195	9,195	9,121	9,121	7,363	9,121	3,097
Clustering	Indiv	Indiv	Indiv	Indiv	Indiv	Indiv	Indiv	Indiv
FE					Country	Country	Country & Period	Country
Proposals	All	All	All	All	All	Accepted Only	All	Periods 11-15 Only
Weights	Country	Country	Country	Country	Country	Country	Country	Country

Notes: *$p < 0.05$, **$p < 0.01$, ***$p < 0.001$. Standard errors clustered at the individual level in parentheses.

Table S8: **Equality, Exclusionary Alliances, and Individual and Country-Level Predictors (with Proposer Weighting)**

	(1)	(2)	(3)	(4)	(5)	(6)	(7)	(8)
**Panel A. Dependent Variable: Equality**								
Female	4.62***			3.23***	3.82***	3.54***	3.88***	4.17***
	(0.60)			(0.62)	(0.59)	(0.60)	(0.58)	(0.75)
High CRT Score		-3.93***		-3.40***	-2.87***	-2.87***	-2.86***	-1.95**
		(0.61)		(0.61)	(0.60)	(0.63)	(0.60)	(0.76)
Political Attitudes Index								
(Right → Left)			3.77***	3.25***	2.44***	2.58***	2.48***	2.49***
			(0.47)	(0.48)	(0.51)	(0.50)	(0.51)	(0.63)
**Panel B. Dependent Variable: Proposed Exclusionary Alliance**								
Female	-0.15***			-0.10***	-0.13***	-0.12***	-0.13***	-0.15***
	(0.02)			(0.02)	(0.02)	(0.02)	(0.02)	(0.02)
High CRT Score		0.17***		0.15***	0.11***	0.10***	0.11***	0.07**
		(0.02)		(0.02)	(0.02)	(0.02)	(0.02)	(0.03)
Political Attitudes Index								
(Right → Left)			-0.08***	-0.07***	-0.07***	-0.07***	-0.07***	-0.08***
			(0.01)	(0.01)	(0.01)	(0.02)	(0.01)	(0.02)
N	9,121	9,195	9,195	9,121	9,121	7,363	9,121	3,097
Clustering	Indiv	Indiv	Indiv	Indiv	Indiv	Indiv	Indiv	Indiv
FE					Country	Country	Country & Period	Country
Proposals	All	All	All	All	All	Accepted Only	All	Periods 11-15 Only
Weights	Proposer	Proposer	Proposer	Proposer	Proposer	Proposer	Proposer	Proposer

Notes: *$p < 0.05$, **$p < 0.01$, ***$p < 0.001$. Standard errors clustered at the individual level in parentheses.

Table S9: **Equality, Exclusionary Alliances, and Individual and Country-Level Predictors (Averages)**

	(1)	(2)	(3)	(4)	(5)	(6)	(7)	(8)
**Panel A. Dependent Variable: Equality**								
Female	4.62***			3.23***	3.82***	3.85***	3.66***	4.06***
	(0.61)			(0.62)	(0.59)	(0.59)	(0.59)	(0.74)
High CRT Score		-3.93***		-3.40***	-2.87***	-2.90***	-3.18***	-2.17**
		(0.61)		(0.61)	(0.61)	(0.61)	(0.61)	(0.75)
Political Attitudes Index								
(Right → Left)			3.77***	3.25***	2.44***	2.43***	2.25***	2.74***
			(0.47)	(0.48)	(0.51)	(0.51)	(0.51)	(0.63)
**Panel B. Dependent Variable: Proposed Exclusionary Alliance**								
Female	-0.15***			-0.10***	-0.13***	-0.13***	-0.13***	-0.14***
	(0.02)			(0.02)	(0.02)	(0.02)	(0.02)	(0.02)
High CRT Score		0.17***		0.15***	0.11***	0.11***	0.11***	0.08***
		(0.02)		(0.02)	(0.02)	(0.02)	(0.02)	(0.03)
Political Attitudes Index								
(Right → Left)			-0.08***	-0.07***	-0.07***	-0.07***	-0.06***	-0.09***
			(0.01)	(0.01)	(0.01)	(0.01)	(0.02)	(0.02)
N	1,470	1,482	1,482	1,470	1,470	1,470	1,465	1,339
Clustering	Indiv	Indiv	Indiv	Indiv	Indiv	Indiv	Indiv	Indiv
FE					Country	Country	Country	Country
Proposals	All	All	All	All	All	All	Accepted	Periods 11-15
Controls						# Times Proposed		

Notes: *$p < 0.05$, **$p < 0.01$, ***$p < 0.001$. Standard errors clustered at the individual level in parentheses.

exclusion increases, as individuals answer more of the three CRT questions correctly, and that differences are statistically distinguishable from zero.

Table S10: **CRT Results with Categorical Operationalization**

	Equality (1)	Exclusion (2)
**# of Correct CRT Answers**		
1/3 Correct	-1.63*	0.07**
	(0.80)	(0.02)
2/3 Correct	-3.86***	0.14***
	(0.80)	(0.03)
3/3 Correct	-6.16***	0.24***
	(0.95)	(0.03)
N	9,195	9,195
Clustering	Indiv	Indiv
FE	Country	Country
Proposals	All	All

Notes: *$p < 0.05$, **$p < 0.01$, ***$p < 0.001$. Standard errors clustered at the individual level in parentheses. Reference category is 0/3 correct CRT answers.

S5.4 Robustness and Discussion of Ideological Orientation Operationalization

In Figure 2 (bottom panel), we display differences in exclusionary behavior by individuals' ideological orientations. For this analysis, we classify an individual as left- or right-leaning depending

on where their political attitudes fell in relation to other respondents *in their country of residence*. We measure an individual's ideological placement relative to other respondents in the same country, rather than globally, for a few reasons. First, we expect that where individuals use ideological orientation as a method to distinguish themselves from others, they will primarily do so by comparing themselves versus fellow citizens and not globally. Second, we anticipate that the perceived ideological spectrum is related more closely to social cleavages, party systems, and historical legacies at the national versus international level. Third, comparing individuals' ideologies to those of individuals across countries would make one's placement highly contingent on the set of countries included in our sample[11]. Fourth, we limit concerns about variations being driven by different understandings of our policy questions, versus different ideological preferences. Nevertheless, we show that our results are robust to using a more absolute (versus relative) measure of ideological orientation: one's political attitudes index score (see Table 1). We also note that results are robust to defining orientation globally, rather than locally (results available upon request).

S6 Additional Results

S6.1 Country-level Results

Tables S11 and S12 show results, including period and session fixed effects, broken down by respondent country for each of our two measures of exclusionary behavior. We find firstly that in no country is the opposite effect than the one reported in the main paper statistically distinguishable from zero: that is, in no country do women, left-leaning, and low cognitive reasoning individuals propose more exclusionary divisions. Second, we observe that the reported pooled sample gender differences are statistically distinguishable from zero for proposal equality and exclusion, respectively, in 11 and 9 of 14 countries; CRT differences in 5 and 6 of 14 countries; and ideological orientation in 7 and 8 of 14 countries.

Table S11: **Proposal Equality by Country**

	Country													
	AUS	AUT	CHN	COL	DNK	EGY	DEU	GTM	JPN	KEN	ESP	GBR	USA	URY
	(1)	(2)	(3)	(4)	(5)	(6)	(7)	(8)	(9)	(10)	(11)	(12)	(13)	(14)
**Panel A. Gender**														
Female	6.52**	4.23	-0.52	8.25***	1.96	6.69**	7.81**	6.95***	4.82*	4.47*	5.28*	7.19**	4.91*	4.57*
	(2.17)	(2.54)	(2.07)	(1.86)	(2.19)	(2.37)	(2.70)	(1.88)	(2.02)	(2.20)	(2.35)	(2.22)	(2.18)	(1.99)
N	651	699	547	660	735	908	559	738	705	603	533	592	564	627
**Panel B. CRT**														
High CRT Score	-2.64	-2.73	2.98	-9.76***	-1.24	-3.78	-4.03	-5.28*	-5.01**	-7.10*	-3.05	-6.78**	-2.80	-7.03***
	(2.28)	(2.45)	(2.17)	(2.36)	(2.57)	(2.73)	(2.71)	(2.17)	(1.89)	(2.89)	(2.40)	(2.38)	(2.17)	(1.82)
N	660	703	547	660	754	908	559	749	705	610	533	595	572	640
**Panel C. Political Attitudes**														
Political Attitudes Index (Right → Left)	2.54	3.68*	-1.74	3.10	4.34*	0.36	3.95	6.01***	4.19*	0.42	5.15**	2.91	5.82***	3.48
	(1.87)	(1.45)	(2.18)	(1.56)	(1.90)	(2.59)	(2.18)	(1.66)	(1.76)	(1.76)	(1.79)	(2.28)	(1.11)	(1.88)
N	660	703	547	660	754	908	559	749	705	610	533	595	572	640
Clustering	Indiv	Indiv	Indiv	Indiv	Indiv	Indiv	Indiv	Indiv	Indiv	Indiv	Indiv	Indiv	Indiv	Indiv

Notes: *$p < 0.05$, **$p < 0.01$, ***$p < 0.001$. Standard errors clustered at the individual level in parentheses. All regressions include session and period fixed effects.

S6.2 Group Composition

Tables S13 and S14 evaluate *accepted* proposal equality and exclusion based on the number of women, left-leaning, and high CRT individuals in a proposer's group. Table S13 shows that proposal equality increases, and exclusion decreases, as there are more women, left-leaning, and low CRT individuals in a group, and that differences are statistically distinguishable from zero. Table S14 displays descriptive results where we compare the mean proposal equality and exclusion for different group compositions.

S6.3 Comparison of Results by Whether Previous Study Conducted in Country

Table S15 compares proposal equality and exclusion based on whether a respondent in our sample was from one of the three countries where previous comparable studies have been conducted (US, UK, or Spain, see Section S7) or not (any of the other 11 countries in our sample). We find that average proposal equality is lower, and exclusion higher, for individuals residing in these three countries as compared to the other 11 in our sample, and that differences are statistically distinguishable from zero.

Table S12: **Proposal Exclusion by Country**

	Country													
	AUS	AUT	CHN	COL	DNK	EGY	DEU	GTM	JPN	KEN	ESP	GBR	USA	URY
	(1)	(2)	(3)	(4)	(5)	(6)	(7)	(8)	(9)	(10)	(11)	(12)	(13)	(14)
**Panel A. Gender**														
Female	-0.19*	-0.09	-0.05	-0.24***	-0.19**	-0.21***	-0.15*	-0.17*	-0.19**	-0.08	-0.27***	-0.27***	-0.14	-0.13
	(0.08)	(0.06)	(0.06)	(0.05)	(0.06)	(0.06)	(0.07)	(0.07)	(0.07)	(0.05)	(0.08)	(0.06)	(0.08)	(0.07)
N	651	699	547	660	735	908	559	738	705	603	533	592	564	627
**Panel B. CRT**														
High CRT Score	0.06	0.05	-0.08	0.35***	0.12	0.20**	0.08	0.21**	0.21**	0.13	0.16	0.17*	0.09	0.29***
	(0.08)	(0.06)	(0.07)	(0.07)	(0.06)	(0.07)	(0.08)	(0.07)	(0.07)	(0.07)	(0.08)	(0.07)	(0.08)	(0.06)
N	660	703	547	660	754	908	559	749	705	610	533	595	572	640
**Panel C. Political Attitudes**														
Political Attitudes Index (Right → Left)	-0.05	-0.11**	0.03	-0.13**	-0.10*	-0.10*	-0.08	-0.16**	-0.13*	-0.03	-0.14*	-0.09	-0.19***	-0.12
	(0.06)	(0.04)	(0.07)	(0.04)	(0.05)	(0.04)	(0.06)	(0.06)	(0.06)	(0.04)	(0.06)	(0.07)	(0.04)	(0.06)
N	660	703	547	660	754	908	559	749	705	610	533	595	572	640
Clustering	Indiv	Indiv	Indiv	Indiv	Indiv	Indiv	Indiv	Indiv	Indiv	Indiv	Indiv	Indiv	Indiv	Indiv

Notes: $*p < 0.05$, $**p < 0.01$, $***p < 0.001$. Standard errors clustered at the individual level in parentheses. All regressions include session and period fixed effects.

Table S13: **Group Composition, Proposal Equality and Exclusion: Accepted Proposals Only**

	(1)	(2)	(3)	(4)
**Panel A. Dependent Variable: Equality**				
# Women in Group	1.36***			1.07***
	(0.31)			(0.32)
# Left-Leaning in Group		1.53***		1.31***
		(0.30)		(0.31)
# High CRT in Group			-1.07***	-0.90**
			(0.30)	(0.30)
**Panel B. Dependent Variable: Proposed Exclusionary Alliance**				
# Women in Group	-0.04***			-0.03***
	(0.01)			(0.01)
# Left-Leaning in Group		-0.05***		-0.04***
		(0.01)		(0.01)
# High CRT in Group			0.06***	0.06***
			(0.01)	(0.01)
N	7,422	7,422	7,422	7,422
Clustering	Indiv	Indiv	Indiv	Indiv

Notes: $*p < 0.05$, $**p < 0.01$, $***p < 0.001$. Standard errors clustered at the individual level in parentheses.

Table S14: **Group Composition, Proposal Equality and Exclusion: Accepted Proposals Only**

	Mean Equality (1)	Mean Exclusion (2)	N (3)
**# Women in Group**			
0	79.06	0.42	999
1	80.14	0.41	2,698
2	81.50	0.35	2,779
3	83.17	0.29	946
**Total**	80.89	0.37	7,422
**# Left-Leaning in Group**			
0	78.70	0.45	1,076
1	80.27	0.39	3,087
2	82.01	0.34	2,595
3	82.95	0.30	664
**Total**	80.89	0.37	7,422
**# High CRT in Group**			
0	82.60	0.28	1,480
1	81.17	0.36	2,547
2	80.14	0.41	2,386
3	79.44	0.46	1,009
**Total**	80.89	0.37	7,422

Table S15: **Comparison of Countries by Whether Previous Study Conducted**

	Equality (1)	Exclusion (2)
**Country Group**		
US, UK, Spain	-2.10**	0.13***
	(0.80)	(0.03)
N	9,195	9,195
Clustering	Indiv	Indiv
FE	Period	Period

Notes: $^{*}p < 0.05$, $^{**}p < 0.01$, $^{***}p < 0.001$. Standard errors clustered at the individual level in parentheses. Reference category is the 11 countries in our sample other than the US, UK, and Spain.

S6.4 Learning and Over-Time Changes

Figure S2 displays average proposal equality (left panel) and exclusion (right panel) by period, split by gender (top panel), CRT score (middle panel), and ideological orientation (bottom panel). We observe significant learning: as individuals play more periods, they tend to engage in more exclusionary behavior. However, individuals of different genders, cognitive reasoning levels, and ideological orientations, tend to learn at similar rates, which accounts for the maintenance of a gap in exclusionary divisions based on these individual-level characteristics even in the presence of learning (albeit to a lesser extent for CRT, as discussed in the main text). A comparison of over-time changes across countries reveals similar patterns: while nearly all countries exhibit more exclusionary behavior in later periods of play, they tend to learn at similar rates – leaving level differences between countries largely intact. Figure S3 displays results, for proposal equality (left panel) and exclusion (right panel), and displaying for each country both the average per period (top panel) as well as the linear relationship between period of play and the outcome (bottom panel).

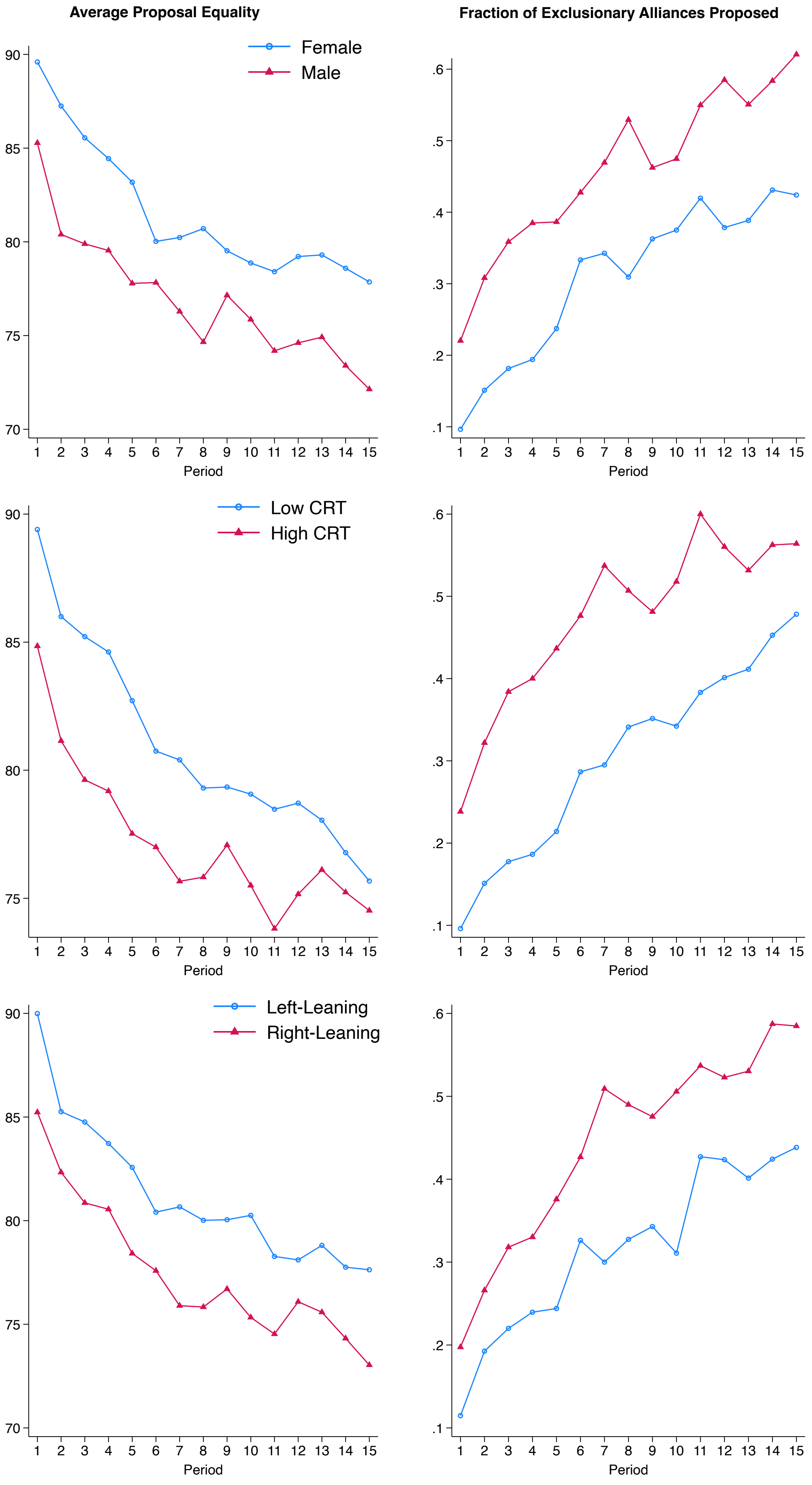

Figure S2: **Learning Across Outcomes and Predictors**

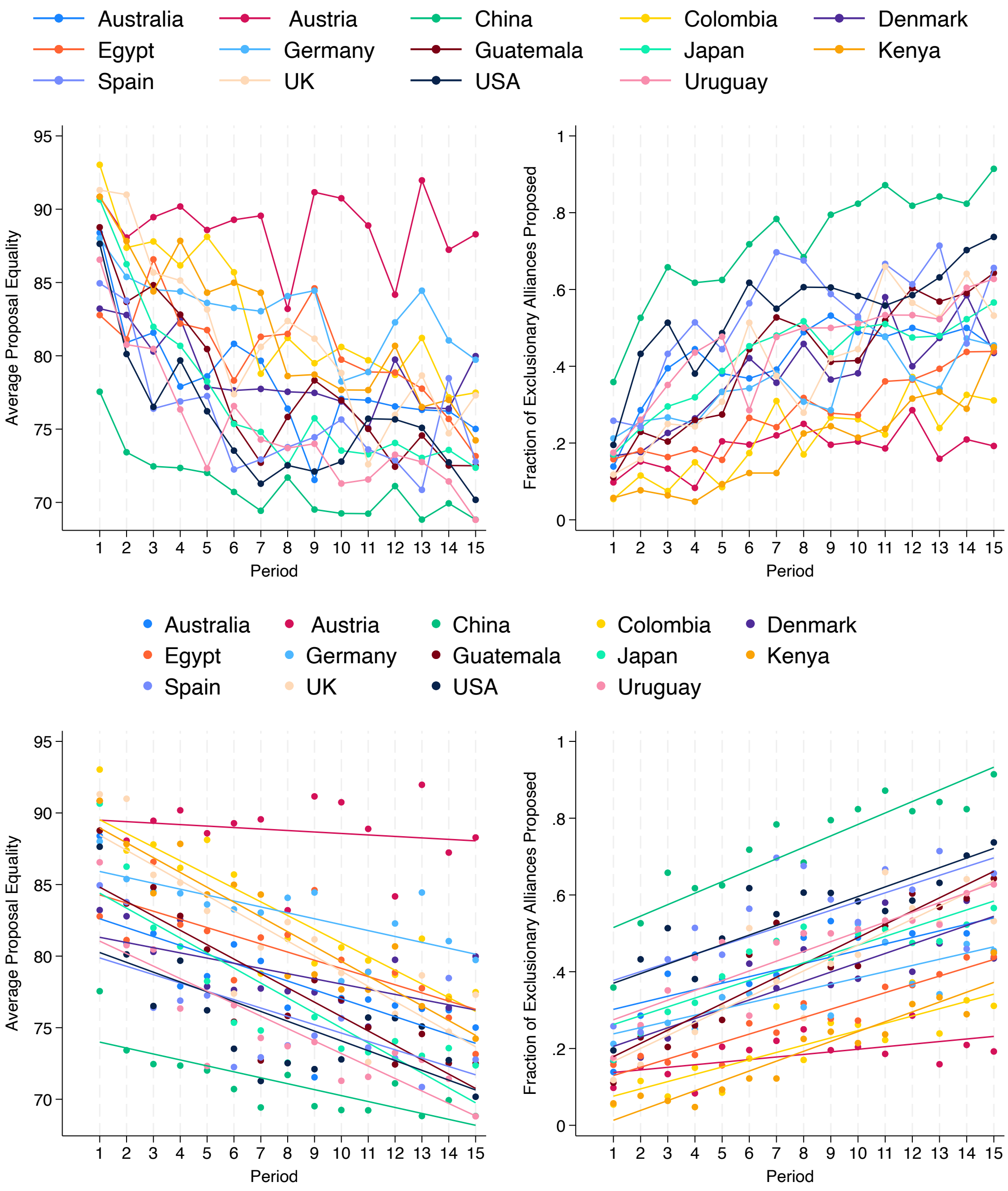

Figure S3: **Learning Across Outcomes and Countries**

Table S16: **Kruskal-Wallis Tests, by Outcome and Period**

	χ^2	p-value
**Panel A. Equality**		
Periods of Play		
All	65.48	0.00
0-3	36.34	0.00
4-6	59.72	0.00
7-9	65.94	0.00
10-12	46.15	0.00
13-15	49.79	0.00
15	45.54	0.00
**Panel B. MWC**		
Periods of Play		
All	60.82	0.00
0-3	37.73	0.00
4-6	52.17	0.00
7-9	55.47	0.00
10-12	49.23	0.00
13-15	50.95	0.00
15	44.33	0.00

Notes: This table shows results from Kruskal-Wallis tests, by outcome (equality or MWC) and period (all, bins of three periods each, or limited to period 15 only). We collapse outcomes by period range and matching group, and we conduct equality-of-populations rank tests by country.

S6.5 Exploring Cultural and Structural Correlations

In this Section, we explore correlations between our outcome measures of exclusionary behavior (proposal equality and the fraction of exclusionary alliances proposed), our measures of cultural hierarchy (our hierarchy tolerance index and its three constitutive components), and a number of cultural and structural measures theorized and found to be of import in the extant literature. First, we detail how these measures were compiled, before presenting results.

Hierarchy Index As detailed in the main text, our hierarchy tolerance index is an inverse-covariance weighted[12] composite comprised of three sources commonly used in cross-cultural analyses. Each composite measure, and its source, is described in more detail below. We selected measures which are both widely used and validated, as well as aligned with our aim of measuring a country's tolerance of hierarchy. Our sources – Hofstede,[13] Schwartz,[14] and the World Values Survey[15] developed by Ronald Inglehart and others – are often among the first cited in cross-cultural studies.[16–19] Importantly, these measures assume that individual-level responses reflect society-wide cultural preferences. As Schwartz (2006, pp.142-143) writes, "I assume that the average value priorities of societal members point to the underlying cultural emphases to which they are exposed (Schwartz, 2004). Like Hofstede (2001) and Inglehart (1997), I therefore infer the cultural value orientations that characterize societies by averaging the value priorities of individuals in matched samples from each society." The measures we use from each source are as follows:

- **Hofstede Power Distance Index (PDI)**[20] This measure, which was compiled by social psychologist Geert Hofstede, was designed – as Hofstede (2010, p.55) writes – to distinguish countries "by the way they tend to deal with inequalities...[PDI] reflects the range of answers found in the various countries to the basic question of how to handle the fact that people are unequal. It derives its name from research...into the emotional distance that separates subordinates from their bosses".[20] As Beugelsdijk and Welzel (2018, p.1473) describe, "Power Distance versus Closeness reflects the extent to which people reject (Distance) or appreciate (Closeness) hierarchies and the authority of a few over the many."[17] The measure is derived from interviews with IBM employees from different countries and concerned their views, beliefs, and behaviors about themselves, as well as the day-to-day reality in which they were embedded. The questions[5] on which the Index is based include aspects related both to hierarchy and decision-making, and thus we expect that the measure will be broad enough to bear relevance for exclusionary behavior as measured both by how equally goods are distributed and the degree of inclusivity of the decision-making process.

- **Schwartz Hierarchy Cultural Dimension**[14] As Schwartz (2006, p. 141) details, "The polar alternative labeled cultural hierarchy relies on hierarchical systems of ascribed roles to insure responsible, productive behavior. It defines the unequal distribution of power, roles, and resources as legitimate. People are socialized to take the hierarchical distribution of roles for granted and to comply with the obligations and rules attached to their roles. Values like social power, authority, humility, and wealth are highly important in hierarchical cultures."[14] As Muthukrishna et al. (2020, p.683) write of the measure, "Hierarchy refers to unequal distribution of power."[19] Schwartz cultural orientations were first validated on samples of

[5]Respondents were asked three questions which form the PDI index. First, "How frequently, in your experience, does the following problem occur: employees being afraid to express disagreement with their managers?" Second, perceptions of how their boss made decisions (autocratic, paternalistic, or neither). Third, respondents' own preferences for their boss's decision-making style (autocratic or paternalistic, or preference for a majority vote).[20]

students (115 samples from 64 countries) and teachers (80 samples from 58 countries) between 1988-2000.[14] Most samples included 180-280 respondents; in total 67 countries and 70 cultural groups were covered, and over 75,000 individuals completed the Schwartz Value Survey (SVS).[14] The measures were subsequently validated on representative national samples from the European Social Survey (ESS).[14] Schwartz computed cultural value orientations using 45 items from the SVS. Respondents were tasked with rating the importance of different values "as guiding principles in MY life."[14] For hierarchy, these values included social power (control over others, dominance), wealth (material possessions, money), authority (the right to lead or command), humble (modest, self effacing), and influential (having an impact on people and events). We take the hierarchy dimension as a measure of a culture's acceptance of hierarchy.

- **World Values Survey (WVS)/European Values Survey (EVS)**[15] For our third measure, we combined two large, cross-national surveys – the WVS and EVS – often used to study cross-cultural differences.[19,21] We use results from the 2017-2022 wave covering all countries in our experiment. We take as our measure responses to a question that asked individuals to state from 1-10 how essential of a characteristic of democracy was it that "people obey their rulers".[22] While we recognize that respondents do not all inhabit democracies and thus may interpret the question differently, research indicates that affirmative responses to the question appear to reflect real beliefs and are often tied to preferences for more hierarchical or authoritarian power structures.[23] As Kirsch and Welzel (2019, 86) conclude, "authoritarian notions of democracy embody a strong belief element where they exist."[23] We ultimately decided that this measure was a better proxy for acceptance of hierarchy and power inequalities than available alternatives.[6]

We first normalized all measures and then combined them into a single, Hierarchy Tolerance Index, using inverse-covariance[12] weighting.[7] As Appendix Figure S4 illustrates, consistent with expectation, our measures of hierarchy are all positively correlated with each other. This is also consistent with research indicating conceptual overlap between Hofstede,[13] Schwartz,[14] and the WVS/EVS[15], regarding the distribution of power in society.[18] Appendix Table S5 indicates that the countries in our sample cover a broad range of values on the hierarchy measures and our overall index.

Further Cultural and Structural Correlates In order to further consider the potential role of culture, generally, as well as hierarchy tolerance, specifically, in explaining our cross-country patterns, we also examined a number of alternative cultural and structural country-level variables factors found to be of import in other cross-national studies. In particular, we used data from exhaustive cross-cultural studies by Awad et al. (2018)[24], Muthukrishna et al. (2020)[19], and Schulz et al. (2019)[25]. We included all possible variables of interest, and we supplemented them as needed (with an indicator for whether a country was a Western, Educated, Industrialized, Rich, and Democratic (WEIRD) country or not, as well as a measure of Purchasing Power Parity (PPP) adjusted mean earnings in our study).

[6] For instance, a respondent saying that "greater respect for authority" in the future would be a good (bad) thing might indicate that they felt there was too little (too much) respect for authority at the current time, as opposed to reflecting their belief that there was a high (low) level at the present time.

[7] Our Hierarchy Tolerance Index, as well as our Hofstede and WVS/EVS measures, include values for all countries, whereas our Schwartz measure includes data on 11 of 14 countries – Guatemala, Kenya, and Uruguay are missing for the Schwartz dataset. Following the literature, we report the average of East and West Germany for our measure of Germany for the Schwartz dataset.

The resultant set of variables cover a wide range of measures: they include data on blood donations per 1,000 individuals as a measure of altruism [19]; data on unpaid tickets of United Nations diplomats as a measure of corruption norms, [19]; data on exposure to the Western Church, which scholars have argued led to less cousin marriage, less powerful kinship-driven institutions, and greater individualism [25]; data on a country's gender gap in health and survival[8] as a measure of gender norms; data on a country's Gini coefficient as a measure of its inequality [24]; and data on a country's degree of rule of law as a measure of its institutional capacity. [24]

We explore the data in a few different ways. First, we display summary statistics by country (Appendix Table S5), which illustrates the high degree of cultural and structural diversity in our sample. Second, we display correlations between our cultural and structural variables, and between these variables and our outcomes (country-level averages in proposal equality and fraction of exclusionary alliances proposed). We do so for most variables in Appendix Figure S4; we do so separately for variables which measure cultural and genetic distance from the United States [19,24] in Appendix Figure S5, as these variables require that all variables be calculated as distance from the U.S. In Appendix Figures S4-S5, red (blue) colors correspond to more negative (positive) correlations, with the size of the circle corresponding to the magnitude of the correlation coefficient; darker colors also reflect larger coefficients, and we also display the correlation coefficient in text inside each cell. Appendix Tables S17-S19 display corresponding correlation coefficients and p-values.

A few observations are notable from these figures. First, we observe that our measures of cultural acceptance of hierarchy are generally the most highly correlated with our observed outcomes.[9] Second, we find that our measures of tolerance of hierarchy are generally correlated with other variables in ways we would anticipate: for instance, greater tolerance of inequality is negatively associated with our proxy for altruism (blood donation) and our measure of rule of law, and it is positively associated with country-level Gini.[10] Taken together, these findings increase our confidence that, in a sample of broad cultural diversity: culture matters, our specific measure of culture (hierarchy tolerance) is of particular relevance in explaining cross-country trends, and our measure of hierarchy tolerance is indeed capturing the intended construct.

Considering Country-Level Gini and Differences from PDI Finally, we consider in more detail one alternative structural explanation of cross-country differences: a country's Gini coefficient. On the one hand, one might imagine that a country's Gini coefficient proxies well for individuals' views on and tolerance of inequality and thus should be a strong predictor of their likelihood of engaging in exclusionary behavior. Indeed, scholars have theorized not only that levels of country-level income inequality might matter, but further that changes in inequality might be expected to lead to shifts in individuals' views on redistributive policy

[8]According to Awad et al. (2018, p.63), the measure is "(a composite in which higher scores indicated higher ratios of female to male life expectancy and sex ratio at birth-a marker of female infanticide and anti-female sex-selective abortion)". [24]

[9]Interestingly, one variable that also exhibits a high degree of correlation is a country's gender gap in health and survival. One possible interpretation is that the variable may pick up acceptance of hierarchy more broadly, as the variable also exhibits correlations with our measures of hierarchy tolerance (see Appendix Table S18). Note, however, that gender gap distance from the U.S. exhibits a weak correlation with our outcomes (see Appendix Figure S5).

[10]These correlation coefficients are all statistically distinguishable from zero, see Appendix Tables S17-S18.

Figure S4: **Country-Level Correlates**

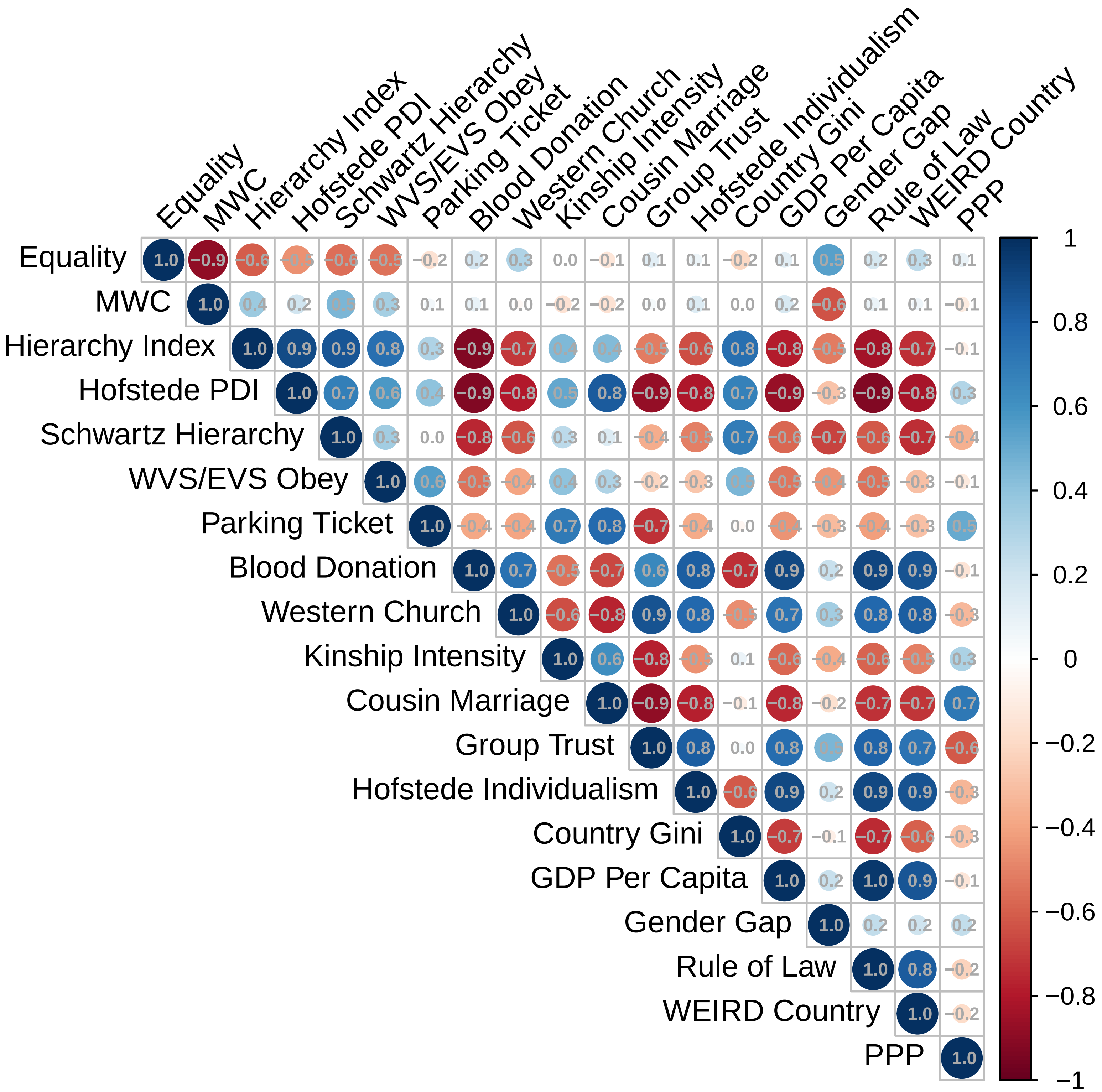

and even their subsequent vote.[11]

[11]See Kenworthy and McCall (2008) for a discussion of the theory underlying an expected link between income inequality and government redistributive policy.[26]

Figure S5: **Country-Level Correlates: Distance from the U.S.**

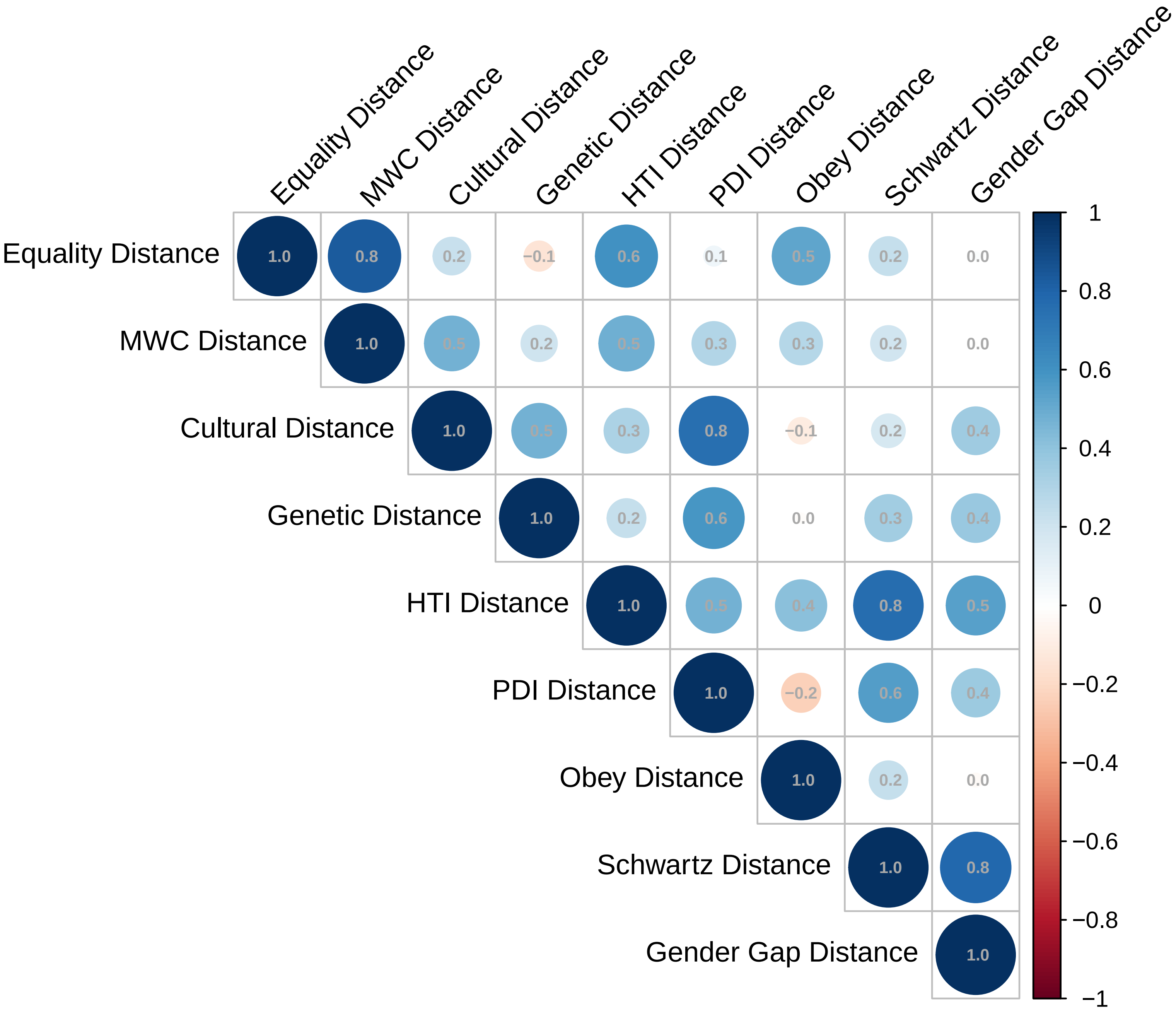

On the other hand, there are both theoretical and empirical reasons to doubt the relevance of country-level Gini as a predictor of cultural attitudes and behaviors regarding exclusion. Theoretically, the expectation of an association between country-level inequality and societal and cultural attitudes and behaviors relies on a number of tenuous links.[26] Notably, a country's level of inequality may not be reflective of its citizens' preferences, but rather of a number of other macro-level factors, from the party system, to the regime type, to historical legacies such as colonialism.[27] Further, individuals may be unaware of the level of inequality in their country or, where they are aware, it may not map cleanly onto their preferred policies

or actions.[26]

Empirically, studies of macro-level inequality and micro-level opinion and behaviors also provide reason for caution. Researchers have reported conflicting findings, for instance, on the link between income dispersion and views on redistribution, with a number of scholars concluding that there does not appear to be an association.[26,28,29] Lupu and Pontusson (2011, p.316) note that many studies have found evidence contrary to their theoretical expectation and highlight in particular the apparent "difficulty of reconciling within- and cross-country evidence".[29] Nor are such findings limited to the issue of views toward redistribution: scholars evaluating the relationship between Gini and outcomes ranging from depression[30] to terrorist risk[31] also find no evidence of an association.

In comparison to Gini and drawing on the above discussion, we expect our measures of hierarchy tolerance to be a stronger predictor of individuals' willingness to engage in exclusionary behavior.[13] First, the measures are derived from *individual* views, beliefs, and behaviors – about oneself, as well as the day-to-day reality in which one is embedded. The measures thus more directly capture individuals' views and behaviors as compared with Gini which, as noted above, relies on a number of tenuous theoretical links from macro-level conditions to individual-level preferences and behaviors. They therefore also allow for a country's culture and preferences to diverge from its structural economic conditions. Second, hiearchy tolerance can be thought of as closer conceptually to the concept of exclusionary behavior studied in this paper. While we expect our measures to pick up views on both to hierarchy and decision-making (see earlier discussion), Gini can be said to be more likely to have views for only one (equality of distribution of resources) and not the other (inclusivity of decision-making) aspect of exclusive behavior we investigate in this paper.

As already shown in Appendix Figure S4, there is only a weak and statistically insignificant correlation (see Appendix Table S17) between country-level Gini and exclusionary behavior in our experiment. This is true despite there being a correlation between Gini and our measures of hierarchy tolerance, indicating that the correlation between hierarchy tolerance and our outcomes is picking up on some other type of cultural variation not captured or driven by country-level Gini. Appendix Figure S6 displays correlations visually and again illustrates the weak correlation between Gini and exclusionary behavior in our experiment.[12] Our analysis thus provides additional evidence in favor of our measure of hierarchy tolerance, as compared with alternative cultural or structural variables.

[12]Interestingly, as might be expected given the above discussion, we observe more support for an association between country-level Gini and our measure of economic exclusion than for our measure of political exclusion. This is potentially consistent with the notion that our measure of hierarchy tolerance more effectively captures a broad undestanding of exclusionary behavior.

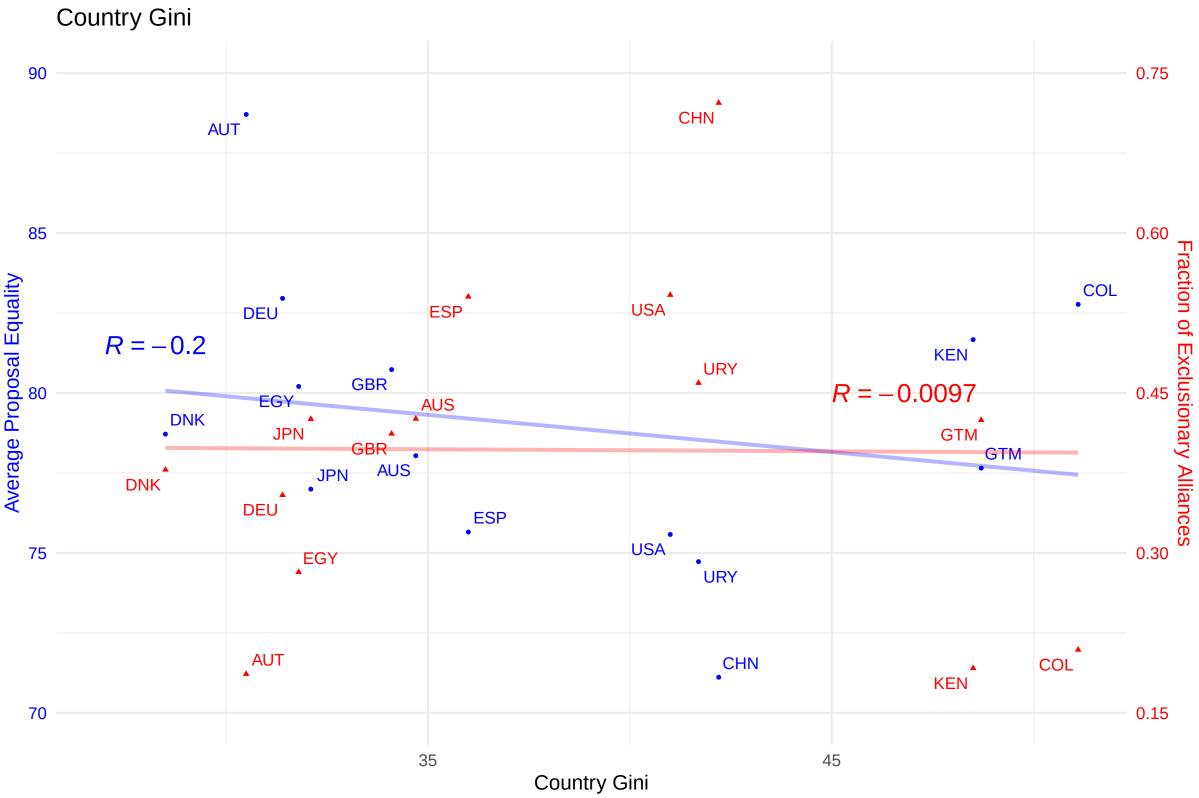

Figure S6: **Equality and Exclusionary Alliances Across Countries: Country Gini**

Table S17: **Correlation Coefficients, Figure S4**

Row	Column	Coefficient	p-value
Equality	MWC	-0,89	0,00
Equality	Hierarchy Index	-0,61	0,02
MWC	Hierarchy Index	0,37	0,20
Equality	Hofstede PDI	-0,45	0,10
MWC	Hofstede PDI	0,20	0,49
Hierarchy Index	Hofstede PDI	0,90	0,00
Equality	Schwartz Hierarchy	-0,55	0,08
MWC	Schwartz Hierarchy	0,46	0,15
Hierarchy Index	Schwartz Hierarchy	0,85	0,00
Hofstede PDI	Schwartz Hierarchy	0,68	0,02
Equality	WVS/EVS Obey	-0,55	0,04
MWC	WVS/EVS Obey	0,34	0,24
Hierarchy Index	WVS/EVS Obey	0,75	0,00
Hofstede PDI	WVS/EVS Obey	0,57	0,03
Schwartz Hierarchy	WVS/EVS Obey	0,35	0,30
Equality	Parking Ticket	-0,16	0,61
MWC	Parking Ticket	0,05	0,86
Hierarchy Index	Parking Ticket	0,31	0,31
Hofstede PDI	Parking Ticket	0,41	0,17
Schwartz Hierarchy	Parking Ticket	0,00	0,99
WVS/EVS Obey	Parking Ticket	0,55	0,05
Equality	Blood Donation	0,18	0,56
MWC	Blood Donation	0,10	0,75
Hierarchy Index	Blood Donation	-0,92	0,00
Hofstede PDI	Blood Donation	-0,92	0,00
Schwartz Hierarchy	Blood Donation	-0,75	0,01
WVS/EVS Obey	Blood Donation	-0,55	0,05
Parking Ticket	Blood Donation	-0,39	0,21
Equality	Western Church	0,31	0,29
MWC	Western Church	-0,03	0,91
Hierarchy Index	Western Church	-0,71	0,00
Hofstede PDI	Western Church	-0,80	0,00
Schwartz Hierarchy	Western Church	-0,63	0,04
WVS/EVS Obey	Western Church	-0,39	0,16
Parking Ticket	Western Church	-0,40	0,18
Blood Donation	Western Church	0,74	0,00
Equality	Kinship Intensity	0,00	1,00
MWC	Kinship Intensity	-0,15	0,60
Hierarchy Index	Kinship Intensity	0,45	0,11
Hofstede PDI	Kinship Intensity	0,52	0,06
Schwartz Hierarchy	Kinship Intensity	0,27	0,42
WVS/EVS Obey	Kinship Intensity	0,40	0,15
Parking Ticket	Kinship Intensity	0,71	0,01
Blood Donation	Kinship Intensity	-0,55	0,05
Western Church	Kinship Intensity	-0,65	0,01
Equality	Cousin Marriage	-0,13	0,72
MWC	Cousin Marriage	-0,16	0,67
Hierarchy Index	Cousin Marriage	0,43	0,21
Hofstede PDI	Cousin Marriage	0,84	0,00
Schwartz Hierarchy	Cousin Marriage	0,15	0,70
WVS/EVS Obey	Cousin Marriage	0,31	0,39
Parking Ticket	Cousin Marriage	0,78	0,01
Blood Donation	Cousin Marriage	-0,66	0,04
Western Church	Cousin Marriage	-0,77	0,01
Kinship Intensity	Cousin Marriage	0,62	0,06
Equality	Group Trust	0,12	0,74
MWC	Group Trust	0,04	0,90
Hierarchy Index	Group Trust	-0,52	0,13
Hofstede PDI	Group Trust	-0,88	0,00
Schwartz Hierarchy	Group Trust	-0,36	0,34
WVS/EVS Obey	Group Trust	-0,22	0,54
Parking Ticket	Group Trust	-0,73	0,03
Blood Donation	Group Trust	0,64	0,04
Western Church	Group Trust	0,86	0,00
Kinship Intensity	Group Trust	-0,78	0,01
Cousin Marriage	Group Trust	-0,88	0,00
Equality	Hofstede Individualism	0,07	0,80
MWC	Hofstede Individualism	0,15	0,61
Hierarchy Index	Hofstede Individualism	-0,65	0,01
Hofstede PDI	Hofstede Individualism	-0,80	0,00
Schwartz Hierarchy	Hofstede Individualism	-0,50	0,12
WVS/EVS Obey	Hofstede Individualism	-0,27	0,34
Parking Ticket	Hofstede Individualism	-0,38	0,20
Blood Donation	Hofstede Individualism	0,83	0,00
Western Church	Hofstede Individualism	0,78	0,00
Kinship Intensity	Hofstede Individualism	-0,45	0,10
Cousin Marriage	Hofstede Individualism	-0,77	0,01
Group Trust	Hofstede Individualism	0,83	0,00
Equality	Country Gini	-0,20	0,49
MWC	Country Gini	-0,01	0,97
Hierarchy Index	Country Gini	0,75	0,00
Hofstede PDI	Country Gini	0,68	0,01
Schwartz Hierarchy	Country Gini	0,69	0,02
WVS/EVS Obey	Country Gini	0,46	0,10
Parking Ticket	Country Gini	-0,04	0,89
Blood Donation	Country Gini	-0,73	0,00

Table S18: **Correlation Coefficients (Continued), Figure S4**

Row	Column	Coefficient	p-value
Western Church	Country Gini	-0,47	0,09
Kinship Intensity	Country Gini	0,08	0,78
Cousin Marriage	Country Gini	-0,09	0,80
Group Trust	Country Gini	0,04	0,91
Hofstede Individualism	Country Gini	-0,61	0,02
Equality	GDP Per Capita	0,12	0,68
MWC	GDP Per Capita	0,17	0,56
Hierarchy Index	GDP Per Capita	-0,79	0,00
Hofstede PDI	GDP Per Capita	-0,86	0,00
Schwartz Hierarchy	GDP Per Capita	-0,58	0,06
WVS/EVS Obey	GDP Per Capita	-0,53	0,05
Parking Ticket	GDP Per Capita	-0,45	0,13
Blood Donation	GDP Per Capita	0,90	0,00
Western Church	GDP Per Capita	0,74	0,00
Kinship Intensity	GDP Per Capita	-0,58	0,03
Cousin Marriage	GDP Per Capita	-0,75	0,01
Group Trust	GDP Per Capita	0,77	0,01
Hofstede Individualism	GDP Per Capita	0,91	0,00
Country Gini	GDP Per Capita	-0,69	0,01
Equality	Gender Gap	0,55	0,04
MWC	Gender Gap	-0,63	0,02
Hierarchy Index	Gender Gap	-0,52	0,06
Hofstede PDI	Gender Gap	-0,29	0,32
Schwartz Hierarchy	Gender Gap	-0,68	0,02
WVS/EVS Obey	Gender Gap	-0,44	0,12
Parking Ticket	Gender Gap	-0,31	0,3
Blood Donation	Gender Gap	0,23	0,46
Western Church	Gender Gap	0,34	0,23
Kinship Intensity	Gender Gap	-0,38	0,18
Cousin Marriage	Gender Gap	-0,17	0,64
Group Trust	Gender Gap	0,46	0,18
Hofstede Individualism	Gender Gap	0,20	0,48
Country Gini	Gender Gap	-0,09	0,77
GDP Per Capita	Gender Gap	0,22	0,44
Equality	Rule of Law	0,17	0,56
MWC	Rule of Law	0,09	0,77
Hierarchy Index	Rule of Law	-0,83	0,00
Hofstede PDI	Rule of Law	-0,92	0,00
Schwartz Hierarchy	Rule of Law	-0,62	0,04
WVS/EVS Obey	Rule of Law	-0,54	0,05
Parking Ticket	Rule of Law	-0,42	0,16
Blood Donation	Rule of Law	0,92	0,00
Western Church	Rule of Law	0,78	0,00
Kinship Intensity	Rule of Law	-0,59	0,03
Cousin Marriage	Rule of Law	-0,72	0,02
Group Trust	Rule of Law	0,80	0,01
Hofstede Individualism	Rule of Law	0,91	0,00
Country Gini	Rule of Law	-0,74	0,00
GDP Per Capita	Rule of Law	0,97	0,00
Gender Gap	Rule of Law	0,24	0,41
Equality	WEIRD Country	0,26	0,37
MWC	WEIRD Country	0,06	0,83
Hierarchy Index	WEIRD Country	-0,73	0,00
Hofstede PDI	WEIRD Country	-0,83	0,00
Schwartz Hierarchy	WEIRD Country	-0,73	0,01
WVS/EVS Obey	WEIRD Country	-0,29	0,31
Parking Ticket	WEIRD Country	-0,30	0,33
Blood Donation	WEIRD Country	0,87	0,00
Western Church	WEIRD Country	0,82	0,00
Kinship Intensity	WEIRD Country	-0,50	0,07
Cousin Marriage	WEIRD Country	-0,72	0,02
Group Trust	WEIRD Country	0,74	0,01
Hofstede Individualism	WEIRD Country	0,87	0,00
Country Gini	WEIRD Country	-0,59	0,03
GDP Per Capita	WEIRD Country	0,86	0,00
Gender Gap	WEIRD Country	0,20	0,48
Rule of Law	WEIRD Country	0,83	0,00
Equality	PPP	0,09	0,75
MWC	PPP	-0,11	0,71
Hierarchy Index	PPP	-0,09	0,77
Hofstede PDI	PPP	0,29	0,31
Schwartz Hierarchy	PPP	-0,36	0,28
WVS/EVS Obey	PPP	-0,12	0,69
Parking Ticket	PPP	0,50	0,08
Blood Donation	PPP	-0,14	0,64
Western Church	PPP	-0,32	0,26
Kinship Intensity	PPP	0,32	0,27
Cousin Marriage	PPP	0,72	0,02
Group Trust	PPP	-0,61	0,06
Hofstede Individualism	PPP	-0,33	0,25
Country Gini	PPP	-0,29	0,32
GDP Per Capita	PPP	-0,14	0,64
Gender Gap	PPP	0,24	0,41
Rule of Law	PPP	-0,22	0,45
WEIRD Country	PPP	-0,18	0,54

Table S19: **Correlation Coefficients, Figure S5**

Row	Column	Coefficient	p-value
Equality Distance	MWC Distance	0,83	0,00
Equality Distance	Cultural Distance	0,22	0,57
MWC Distance	Cultural Distance	0,47	0,20
Equality Distance	Genetic Distance	-0,14	0,65
MWC Distance	Genetic Distance	0,20	0,50
Cultural Distance	Genetic Distance	0,47	0,20
Equality Distance	HTI Distance	0,61	0,03
MWC Distance	HTI Distance	0,48	0,10
Cultural Distance	HTI Distance	0,32	0,41
Genetic Distance	HTI Distance	0,24	0,44
Equality Distance	PDI Distance	0,06	0,84
MWC Distance	PDI Distance	0,30	0,32
Cultural Distance	PDI Distance	0,75	0,02
Genetic Distance	PDI Distance	0,58	0,04
HTI Distance	PDI Distance	0,48	0,10
Equality Distance	Obey Distance	0,53	0,07
MWC Distance	Obey Distance	0,29	0,34
Cultural Distance	Obey Distance	-0,11	0,78
Genetic Distance	Obey Distance	-0,02	0,96
HTI Distance	Obey Distance	0,41	0,16
PDI Distance	Obey Distance	-0,24	0,43
Equality Distance	Schwartz Distance	0,24	0,51
MWC Distance	Schwartz Distance	0,20	0,59
Cultural Distance	Schwartz Distance	0,18	0,71
Genetic Distance	Schwartz Distance	0,35	0,33
HTI Distance	Schwartz Distance	0,77	0,01
PDI Distance	Schwartz Distance	0,55	0,10
Obey Distance	Schwartz Distance	0,23	0,52
Equality Distance	Gender Gap Distance	0,01	0,97
MWC Distance	Gender Gap Distance	0,00	1,00
Cultural Distance	Gender Gap Distance	0,36	0,34
Genetic Distance	Gender Gap Distance	0,37	0,21
HTI Distance	Gender Gap Distance	0,55	0,05
PDI Distance	Gender Gap Distance	0,37	0,22
Obey Distance	Gender Gap Distance	-0,03	0,93
Schwartz Distance	Gender Gap Distance	0,79	0,01

S6.6 Stated and Revealed Preferences

In addition to measuring exclusionary tendencies behaviorally, we also asked individuals directly about their views on engaging in exclusion. Specifically, after the study had concluded, we presented them with scenarios where we asked them to imagine that a group of three individuals was dividing a sum of money and that at least two of the three needed to agree on a split; we then asked them their views on the possibility of a two-way split occurring (with the third person receiving nothing). We asked respondents four versions of this question, varying whether they were sharing their own views regarding acceptability of a two-way split or the likelihood of such a split occuring in their country, as well as whether the amount divided was "earned" or a "windfall". As an example, our wording for the earned, own view acceptability measure was as follows:

> Consider the following situation: A group of three people are negotiating how to split a sum of money. At least two of them must agree on the split. In your view, how acceptable is it to split the money only between two people, with the third person getting nothing? (*1=completely unacceptable, 7=completely acceptable*)

Below, we present results. Findings should be considered suggestive: given that we only measure stated preferences post-treatment, associations with revealed preferences could also be the result of individuals' desires to rationalize, ex post, their previous behavior in the bargaining game. However, we felt it was better to measure such attitudes after the game rather than before, so as to avoid priming individuals to consider fairness or norm-based considerations. We also are missing data on these questions for four of our 14 countries, owing to our decision to add them as the data collection process was ongoing.[13] Table S20 shows the association between the different measures of stated preferences and the fraction of times an individual proposed an exclusionary alliance. We observe an association between all four stated preference measures and our revealed preference measure of exclusionary behavior. The largest coefficient corresponds to an individual's own views on the acceptability of a two-way split.

To gain additional insights into the relationship between stated and revealed preferences, in Figure S7 we illustrate how we would expect the fraction of exclusionary alliances proposed to vary with an individual's views on the acceptability of the practice. As for the regression output displayed in Table S20, our estimates are based on a specification that includes individual-level predictors (gender, CRT score, and political attitudes index), clustering at the individual level, and country fixed effects. The figure illustrates again an association between stated and revealed preferences; however, we can also see that differences in revealed behavior sometimes appear to be negligible across varying values of stated preferences (1 versus 2 or 3, or 4 versus 5, for example). In sum, then, it appears that while there is a relationship between stated and revealed preferences, the former are not sufficient to fully predict the latter.

[13]The four countries are Colombia, Denmark, Spain, and the United States.

Table S20: **Stated and Revealed Preferences: Exclusionary Alliances**

	DV: Fraction, Exclusionary Alliances Proposed			
	(1)	(2)	(3)	(4)
Acceptability (Self)	0.06***			
	(0.01)			
Likelihood (Others)		0.03***		
		(0.01)		
Acceptability (Self, Bonus)			0.04***	
			(0.01)	
Likelihood (Others, Bonus)				0.02**
				(0.01)
Female	-0.10***	-0.12***	-0.12***	-0.13***
	(0.02)	(0.02)	(0.02)	(0.02)
High CRT Score	0.08***	0.10***	0.10***	0.10***
	(0.02)	(0.02)	(0.02)	(0.02)
Political Attitudes Index				
(Right → Left)	-0.04*	-0.06***	-0.04*	-0.05**
	(0.02)	(0.02)	(0.02)	(0.02)
N	1,072	1,072	1,072	1,072
Clustering	Indiv	Indiv	Indiv	Indiv
FE	Country	Country	Country	Country

Notes: *$p < 0.05$, **$p < 0.01$, ***$p < 0.001$. Standard errors clustered at the specified level in parentheses.

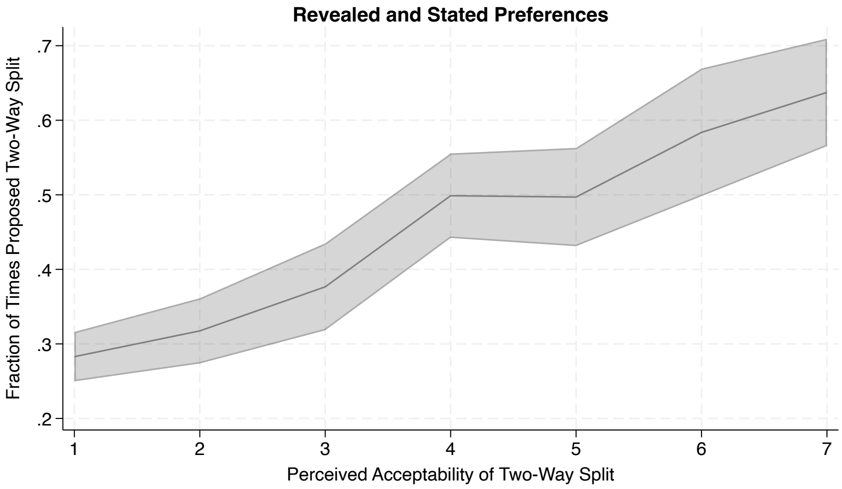

Figure S7: **Revealed versus Stated Preferences**

S7 Mini Meta-Analysis

In this section we analyze the raw data from three-player Baron-Ferejohn [6] experiments that have been published up to 2023. Our goal is two-fold. First, we wish to highlight the skewed view that previous studies had shed of exclusionary behavior, and why a broader cross-cultural study can substantially expand our understanding of human behavior. Second, we want to verify if the behavior in previous experiments is in line with our experiment (for the same countries) in order to rule out experimental design differences as a driver of behavioral differences if any.

We gathered our data primarily from authors' websites when it was publicly accessible. If the data were not publicly available, we reached out directly to the corresponding authors, all of whom generously provided their data. To ensure a complete data set, we conducted an exhaustive search in major academic digital repositories using the keywords "multilateral bargaining experiments" and "Baron and Ferejohn (1989)". In this search effort, we enlisted the help of two research associates to assist with both the search task and the data harmonization process.

We narrowed our study sample to treatments that yielded equivalent insights in terms of the game-theoretic equilibrium payoff distributions to our current experiment. Readers without specialized knowledge on the details of the game-theoretic equilibrium derivations in this game are referred to [6] and [8] for details. More specifically, we ensured that the stationary subgame perfect equilibrium vector of payoff coincided with ours. That is, in expectation every player receives 1/3 of the fund to divide, prior to any person being selected as the proposer. Furthermore, we focus on games without communication in which the fund to distribute is exogenously endowed by the experimenter. Our selection criteria mean that we have chosen treatments characterized by the following parameter configurations:

1. There are three players in a group and the majority voting rule is in place.
2. Equal Real Bargaining Power: In all cases, every member must have an equivalent equilibrium probability of inclusion in a winning coalition. This criteria excludes treatments where some players wield disproportionate voting influence, such as the Apex treatment [32] and the veto treatments [33,34].
3. Symmetric Disagreement Values: In cases where bargaining reaches its final round or breaks down, we require that all players receive identical payoffs.
4. There are at least two rounds of bargaining allowed within each game.
5. The fund available for distribution is determined exogenously.
6. All proposals and voting decisions are made by human subjects rather than computer algorithms.
7. There is no communication between subjects in the game.
8. Subjects have not experienced the same game before. This excludes *repeat subjects* and treatments where subjects played a similar game before the game of interest as in some sessions of one study [35].
9. If the game is played several times, we require that rematching be random and anonymous so that no reputation is built.

A number of studies were included. [33–41] Table S21 summarizes the data, the proportion of exclusionary alliances, and mean equality. By and large, the previous results from the United

Stages, United Kingdom, and Spain our current experiment are quite close to those from our study.

Country	# of Studies	# of Subjects	Proportion of Exclusionary Alliances	Mean Equality
**Previous Studies**				
United States	4	235	0.59	0.71
United Kingdom	4	167	0.41	0.78
Spain	1	16	0.59	0.72

Table S21: **Results from Previous Experimental Studies, 3-player Games**

In addition, there are three other studies with treatments that fit our description, except that they have groups of 5 players.[32,42,43] All these studies were conducted in the United States, and the proportion of exclusionary alliances (i.e. 3 members receive a positive share and 2 receive 0) is 0.59, matching exactly the proportion in 3 player games. The equality is lower, at 0.63. But this is strictly mechanical because in groups of 5 an exclusionary alliance leaves 2 players with a share of 0 (40% of the group) while in groups of 3 it leaves only one player with 0 (33% of the group).

S8 Deviations from Pre-Analysis Plan

We pre-registered a larger project on social preferences and bargaining which can be found in Appendix Section S9. We first note that all empirical expectations presented in this paper were pre-registered. However, because this paper is part of a larger research project – and because we face space constraints – we do not report all pre-registered analyses here. Of course, we are amenable to adding any further analyses that reviewers or editors request.

Other minor deviations include:

1. For presentational reasons and to facilitate comparison with gender, we dichotomized our pre-registered indices measuring cognitive reflection and ideological orientation as displayed in Figure 2 and explained in the main text.
2. Though we pre-registered our intent to probe robustness to learning via period fixed effects – and we include these results in the supplementary materials – we did not pre-register the binned approach displayed visually in Figure 4. However, as above, we elected to include the analyses for presentational purposes.
3. For consistency, we estimate OLS models, including for dichotomous variables (and not probit regression models). Results are robust to probit estimation.
4. Owing to our relatively small cross-country sample size (N=14), we only present correlational analyses of cross-country predictors and treat these analyses as exploratory. We also explore additional cross-country correlates based on reviewer feedback.

S9 Pre-Analyis Plan

Our pre-analysis plan (PAP) was registered in two stages with Open Science Framework.[14] Below, we reproduce a version in its entirety.[15]

S9.1 Study Information

Hypotheses
We expect to observe comparatively more other-regarding behavior in legislative bargaining (and thus for outcomes to stray further from equilibrium prediction):

1. In countries with lower levels of inequality
2. Among individuals who are native to those countries, and who have spent more time, or invested more heavily (for example, by completing the citizenship naturalization process), in those countries
3. Among individuals who express more concerns about inequality, and who are more trusting
4. Among women, as compared with men
5. Among those who are less cognitively sophisticated, that is, who score lower on a series of cognitive reflection tasks
6. We also expect that between-country differences will be magnified where the sample pool is limited to natives of the country, and participants are made aware of this fact

We operationalize other-regarding behavior in three primary ways:

1. Minimum winning coalition: did a participant propose a coalition with the minimum number of players (in our study, one) needed to constitute a simple majority and thus pass a proposal? Or, alternatively, did they choose to include all partners (in our study, two) in the coalition even though they only needed one vote for the proposal to pass?
2. Inequality: how equal was the proposed bargain between partners? In particular, what share of the total budget amount did the participant propose for themselves? For this purpose, we will compute the gini coefficient implied by division of the budget.
3. Voting concerns: when subjects cast their vote, do they focus solely on their share of the pie, or does the inequality of the distribution matter for them?

Regarding the hypotheses stated above, we expect stronger effects with regard to minimum winning coalitions than with regard to inequality (or proposed own share of the budget).

[14] An initial PAP was registered after pilot sessions had been conducted in Denmark. Our addendum added plans for data collection in additional countries, as well as further analyses (in particular, across countries); it was filed after data had been collected in Denmark, Spain, and the U.S. (also Colombia, but prior to the authors accessing this data).

[15] We note where initial and addendum PAPs differed in content. Where no note is made, registrations were identical or the addendum referred to content in the initial PAP submission.

S9.2 Design Plan

Study type
Experiment - A researcher randomly assigns treatments to study subjects, this includes field or lab experiments. This is also known as an intervention experiment and includes randomized controlled trials.

Blinding
For studies that involve human subjects, they will not know the treatment group to which they have been assigned.

Study design
Our experimental implementation of the Baron-Ferejohn game closely follows several other studies[44]. Subjects are grouped in triads and are tasked with deciding how to divide a fixed, divisible resource (hereafter referred to as a pie; in our study, approximately USD $30) by making proposals and voting. In the first period of bargaining, one player in each group is randomly chosen to propose a division of the pie and thereafter everyone votes. If two or more members (including the proposer) vote in favor of the proposal, the result binds, payoffs are realized, and bargaining ends. If not, the process repeats itself until approval. This procedure reflects the closed-amendment rule, in which proposals are voted up or down without a possibility to amend. Whereas in some studies, the pie shrinks by some discount factor with each rejected proposal, in our version there is no discounting.

Subjects will play 15 games and will be randomly rematched after each agreement. Only 2 of the 15 games will be selected at random for payment. Following the completion of the 15 games, subjects will answer a series of questions in which we measure variables concerning our hypotheses under study.

Randomization
We randomize along a few different dimensions:

1. Each participant is randomly assigned to a group of three participants at the start of each period of bargaining. We have been running sessions with 15 periods of play, and we have been using matching groups of no fewer than 9 participants such that individuals are randomly assigned new partners from within this subset of the larger session (in cases where there are 18 or more participants).
2. In each round of bargaining in a given period, one member of each group is randomly selected to be the proposer and thus to propose a division of the $30 between the members of the group. The other two members of the group do not make a proposal but decide whether or not to vote in favor of the proposal. The proposer is automatically counted as voting in favor of the proposal, meaning that only one voter is required to vote in favor of the proposal for it to pass.

S9.3 Sampling Plan

Existing Data
Registration prior to creation of data

Explanation of existing data (initial PAP submission)[16]

We are filing this registration:

1. After conducting sessions of this experiment in Denmark, and analyzing early results (sessions in Denmark are ongoing).
2. After analyzing differences in outcomes between countries using raw data from all published three-player Baron-Ferejohn experiments which includes data from the United States, United Kingdom, and Spain.

   While useful, it is important to note that data from other Baron-Ferejohn studies does not allow us to investigate our hypotheses on the role of political views, gender, or cognitive sophistication in bargaining because these variables were either not collected or unreported in previous studies. Importantly, previous studies also have some different design features for which it is necessary to account.
3. Prior to conducting planned experiment sessions in Spain; b) prior to additional experiment sessions in Denmark, as they are ongoing; and c) prior to conducting sessions in the United States. As we are filing this registration prior to conducting any sessions in Spain and the United States, we are completely unaware of any patterns or summary statistics that may ultimately appear in such data. In contrast, we are aware of potential patterns that may appear in our data from Denmark, as we have thus far obtained approximately half of our desired final sample.

Explanation of existing data (addendum PAP submission)

This addendum concerns data collection in countries including the United Kingdom, Kenya, Guatemala, Egypt, Colombia, China, Japan, and possibly beyond (depending on funding): notably, the United Arab Emirates, Uganda, Mexico, Australia, Germany, and India, as well as more countries if possible. Data has yet to be collected in all of these countries with the exception of Colombia; however, in this single case, the authors still have not accessed the data.

Our original, published pre-registration concerned data collection in Denmark, Spain, and the United States. This data collection has been completed.

Data collection procedures

Initial PAP Submission We will conduct lab experiment sessions in Spain through the Laboratory for Research in Behavioural Experimental Economics (LINEEX, `https://www.lineex.es/en/home/`) at the University of Valencia. In Denmark, we will conduct lab experiment sessions at the Cognition and Behavior Lab (COBE, `https://bss.au.dk/en/cognition-and-behavior-lab`) at Aarhus University. For the United States sample, we plan to do so at the Experimental Economics Laboratory (`https://econlabcal.econ.ohio-state.edu/orsee/public/`) at Ohio State University. Raw data from published studies was obtained with authors' consent. Finally, we may run online sessions of our study to probe the external

[16] As noted in the introduction to this section, our pre-registration was conducted in two stages.

validity of our findings using OTree and recruiting participants either from Amazon.com's Mechanical Turk or using an online panel service, such as Lucid.

For all lab experiment sessions, our only conditions for participation are that a) participants be currently enrolled as students; b) participants be over 18 years of age; c) participants have not already participated in the study; d) participants meet the COVID-19 requirements in the lab (for example, providing proof of vaccination where required); e) that participants provide their informed consent to participate in the study; and f) that participants are proficient in the language of the study. Participants in our lab experiments receive a notification when new experiment sessions are available and sign up to participate if they are interested. We hope to complete our Spain and United States experiment sessions in the Spring of 2022.

Addendum PAP Submission To collect data in additional countries, we have partnered with different experimental laboratories around the world. We are either traveling to conduct the sessions ourselves or hiring research assistants to conduct sessions on our behalf. We have thus far finalized plans (or in the case of Colombia, completed sessions) for sessions at laboratories based at the following universities/institutions (countries):

1. The University of Nottingham (CeDEx lab, United Kingdom)
2. Busara Center for Behavioral Economics (Kenya)
3. Universidad Francisco Marroquín (Guatemala)
4. Universidad del Rosario (Colombia)
5. The British University in Egypt (EBEL lab, Egypt)
6. Shanghai Jiao Tong University (Smith Experimental Economics Lab, China)
7. Kansai University (RISS, Japan)

In addition, we have been in contact with laboratories at the following institutions: New York University Abu Dhabi (United Arab Emirates), Ashoka University (India), and the University of Hamburg (Germany). We are further seeking to expand our sample and may reach out to additional experimental laboratories in the future.

Sample size

We will aim to collect data from approximately 100-120 participants in each country. Each participant will complete 15 periods of play (in groups of 3). Power analyses using data from Denmark and raw data from existing Baron-Ferejohn studies indicate that we will be sufficiently powered to detect effect sizes of interest. In addition, we may report minimum detectable effect sizes after our data has been collected.

S9.4 Variables

Manipulated variables

An individual is either randomly assigned to be a proposer (1) or not (0) in a given period of play.

Measured variables

Initial PAP Submission We have two central dependent variables:

1. Minimum Winning Coalition: equals 1 if a proposer proposes a non-zero share of the pie to two of three members of the group, which corresponds to the number of group members required for a passing vote. Equals zero for any other division. Note that we may adjust the non-zero amount to exceed a "pittance" amount instead of strictly considering it to be greater than zero.
2. Our measure of inequality is the absolute value of 100 - the Gini index value.

In addition to these two central dependent variables, we will also consider the following as dependent variables:

3. Proposer's share as percentage of the total fund.
4. Accepted: equals 1 if a proposal received the minimum number of votes required to pass, 0 otherwise. We may also consider as a discrete variable the number of votes a proposal received (1, 2, or 3).
5. Delay: equals 1 if there were two or more rounds of bargaining (meaning that the round 1 proposal did not pass), 0 otherwise. We may also consider a discrete variable corresponding to the number of rounds of bargaining.
6. All-way split: equals 1 if all three members of the group were proposed a non-zero share of the pie, 0 otherwise.
7. Voting decision: "yes" or "no" is recorded for every non-proposing subject.

We will consider a number of independent variables:

1. Country in which a study was conducted. Options: Denmark, United States, Spain, and (if using external data) the United Kingdom.
2. Demographics of theoretical interest: Gender (male or female, assuming an insufficient number of non-binary participants), proxies for exposure to/investment in the country in which a study was conducted (for example, citizenship) as well as for exposure to other country norms (country of birth, parents' countries of birth).
3. An index of scores on three cognitive reflection tasks. Ranges from 0 to 3, where each correct answer is given a score of 1 and each incorrect answer is given a score of 0.
4. Answers to survey questions meant to measure participants' political opinions on several issues, including: a.) Placement on 10 point scale, from (1) People who are unemployed ought to take any offered job to keep welfare support to (10) People who are unemployed ought to be able to refuse any job they do not want. b.) Placement on 10 point scale, from (1) Competition is good to (10) Competition is damaging. c.) Placement on 10 point scale, from (1) The income distribution ought to be more equal to (10) There ought to be more economic incentive for the individual to work harder. d.) Placement on 10 point scale, from (1) More public companies ought to be privatized (10) More companies ought to be state-owned. We may construct an index of political identity (left - right) based on the answers to these questions.

Addendum PAP Submission Below, we detail variables underlying our additional analyses and, where relevant, associated empirical expectations for the four areas of exploration specified in our addendum description.

1. Cross-country Analysis

   We had previously pre-registered our expectation that in countries with higher economic inequality (as measured by the Gini coefficient) we would expect more unequal sharing of the surplus. We now expand our analysis by considering other potential explanatory variables such as:

   a.) Cultural differences using the Hofstede indicator of Power Distance. We expect that societies that are less tolerant towards power asymmetries share the surplus more equally.

   b.) Cultural differences using the Hofstede index of Individualism vs Collectivism. We expect that societies that are less individualistic share the surplus more equally.

   c.) Welfare spending. We expect more equal sharing in countries with higher welfare spending per capita.

2. Expanded Gender Analysis

   A recent study (Brenøe et al. 2022) has proposed and tested a single item continuous gender identity measure (self-assessed masculinity vs. femininity on a unidimensional 11-point scale) in order to understand if it can have further explanatory power over binary measures (male/female). As part of our exploratory analysis, we will investigate if self-placement on the scale has an effect on bargaining outcomes beyond the effect estimated using a binary gender measure.

   Specifically, we will conduct a similar analysis as in Brenøe et al. (2022): first including only binary gender and only the continuous measure as independent variables in regressions, and then including gender (binary) and the continuous measure together to assess especially if the continuous measure holds additional explanatory power beyond the binary measure.

   Following Brenøe et al. (2022), we will ask respondents to respond to the following question which provides a continuous measure of gender identity:

   "In general, how do you see yourself? Where would you put yourself on this scale (0-10) from "Very masculine" to "Very feminine"? Please indicate your response below."

3. Bargaining Process

   In our original pre-registration we focused on bargaining outcomes (distribution of the fund) and voting behavior. We expand our proposed analysis to include an investigation of the bargaining process. As demonstrated by Baranski and Morton (2021), subjects typically engage in retaliation against failed proposers, meaning that the history of bargaining behavior is central in determining outcomes. Specifically, we will measure retaliatory behavior against a former proposer upon a failed agreement in the following ways: (1) expected payoff in bargaining round "t" for the proposer and voter (separately) from round "t-1", and (2) the same for the probability of inclusion in a minimum winning coalition.

   With respect to the bargaining process and retaliation, we also ask subjects the following questions:

   a.) "If I feel that someone has wronged me, I will retaliate if given the possibility to do so." To what extent do you agree with the previous statement, where 1 means strongly disagree and 7 strongly agree?

   b.) "In general, people in this country retaliate when they feel someone has wronged them if given the possibility to do so." To what extent do you agree with the previous statement, where 1 means strongly disagree and 7 strongly agree?

4. Non-incentivized Measures of Bargaining Behavior
   A secondary goal of our research agenda is to later establish whether or not there is a correlation between students' revealed bargaining behavior and the stated, hypothetical bargaining behavior of representative samples.

   To first compare stated and revealed behavior among our student convenience samples, we ask the following non-incentivized questions about bargaining outcomes and division of the surplus:

   1.) Consider the following situation: "A group of three people are negotiating how to split a sum of money. At least two of them must agree on the split."
      a.) In your view, how acceptable is it to split the money only between two people, with the third person getting nothing? 1 is completely unacceptable, 7 is completely acceptable.
      b.) If three people in this country were to find themselves in this situation, how likely is it that the money will be split only between two of them, with the third person getting nothing? 1 is extremely unlikely, 7 is extremely likely.
   2.) Consider the following situation: "A group of three members of a company's board are tasked with negotiating how to split a sum of "bonus" money. At least two of them must agree on the split."
      a In your view, how acceptable is it to split the money only between two people, with the third person getting nothing? 1 is completely unacceptable, 7 is completely acceptable.
      b If three people in this country were to find themselves in this situation, how likely is it that the money will be split only between two of them, with the third person getting nothing? 1 is extremely unlikely, 7 is extremely likely.

Additional Exploration. In addition to the above exercises, we will also conduct exploratory analyses into possible relationships between bargaining behavior and individuals' a) risk attitudes and b) views on the determinants of success. Specifically, we will ask:

1. Risk. We will ask respondents to answer, on a scale from 0 to 10, the following question: "How willing are you to take risks, in general?"
2. Views on determinants of success. We will ask respondents to answer, on a scale from 1 to 10, the following question: "How would you place your views on this scale? 1 means you agree completely with the statement on the left; 10 means you agree completely with the statement on the right; and if your views fall somewhere in between, you can choose any number in between." We expect more equal sharing among individuals who agree more that success is the result of luck and connections. We also ask this question to compare our sample to representative samples in the World Values Survey.

   Left statement: In the long run, hard work usually brings a better life.

   Right statement: Hard work doesn't generally bring success – it's more a matter of luck and connections.

References:

1. Brenøe, A. A., Heursen, L., Ranehill, E., & Weber, R. A. (2022, May). "Continuous Gender Identity and Economics." In AEA Papers and Proceedings (Vol. 112, pp. 573-77).

2. Baranski, Andrzej, and Rebecca Morton. "The determinants of multilateral bargaining: A comprehensive analysis of Baron and Ferejohn majoritarian bargaining experiments." Experimental Economics (2021): 1-30.

Indices

Our cognitive reflection index is a simple additive index consisting of the sum of scores, where 1 is a correct answer and 0 is an incorrect answer, across the three cognitive reflection tasks.

We may construct an index of political identity (left - right) based on the answers to the questions on political views. We would weigh each equally.

S9.5 Analysis Plan

Statistical models

We will estimate effects using simple OLS regressions for continuous dependent variables (e.g. equality of distribution) and probit regressions for dichotomous variables, with clustering at the individual subject level, as well as two-sample tests of means and proportions. We will probe robustness of findings to including session and period fixed effects. We now return to the hypotheses specified earlier and detail how we will test each. We expect to observe comparatively more other-regarding behavior in legislative bargaining (and thus for outcomes to stray further from equilibrium prediction):

1. In countries with lower levels of inequality.

   To examine expectation 1, we will compare mean outcomes across different countries (Denmark, Spain, the United States, and potentially the United Kingdom) using OLS regression estimations and t-tests. We expect other-regarding behavior to be highest in Denmark and Europe, and lowest in the United States.

2. Among individuals who are native to those countries, and who have spent more time, or invested more heavily (for example, by completing the citizenship naturalization process), in those countries.

   To examine expectation 2, we will compare mean outcomes across the different independent variables (such as citizenship) using OLS regression estimations and t-tests. We expect individuals who are native to countries or who have invested more in those countries to conform more to group behavior in the country in which the session was conducted.

3. Among individuals who express more concerns about inequality, and who are more trusting.

   To examine expectation 3, we will compare mean outcomes across the different independent variables (such as attitudes on inequality) using OLS regression estimations and t-tests.

4. Among women, as compared with men.

   To examine expectation 4, we will compare mean outcomes across participant genders using OLS regression estimations and t-tests.

5. Among those who are less cognitively sophisticated, that is, who score lower on a series of cognitive reflection tasks.

   To examine expectation 5, we will compare mean outcomes across cognitive reflection index scores using OLS regression estimations and t-tests.

6. Between-country differences will be magnified where the sample pool is limited to natives of the country.

   To examine expectation 6, we will compare mean outcomes across session conditions (all natives or not, if these sessions are conducted) using OLS regression estimations and t-tests.

For the study of voting decisions, we will conduct probit regressions (clustering standard errors at the subject level). The independent variables in our regression analysis will be own share received, one variable that captures the fairness of an allocation (gini, proposer's share, or whether the allocation is a three-way split). We will control for gender and cognitive ability. We will estimate the models for each sample (country) separately. To compare behavior across countries, we will pool the data and interact the variable that captures fairness with country indicator variables, while controlling for gender and cognitive ability.

Transformations

See description of variables.

Inference criteria

We will be using two-tailed tests, though we may also report one-tailed tests for directional hypotheses. We will use $p = 0.05$ as our central cutoff though we may note where a p-value is greater than 0.05 but less than 0.10.

Data exclusion

N/A

Missing data

N/A

S10 Acknowledgements

We partnered with the following laboratories to conduct our research. We are grateful for their support in renting or sharing their labs and resources.

1. **Lakelab at the University of Konstanz.** We thank Urs Fischbacher and the lab's team including Regina Stump and Eva Hermann for their support.
2. **Centro Vernon Smith de Economía Experimental at the Universidad Francisco Marroquín in Ciudad de Guatemala.** We thank Susette España and the lab's team, including Francisco Lemus for their support.
3. **Research Instititute for Socionetwork Strategies (RISS) at Kansai University.** We thank Kazuhito Ogawa and his team, including Young Rok and the secretarial office for their support.
4. **Laboratorio de Economía Experimental de la Universidad de Montevideo.** We thank Marcelo Caffera and his team, including Juan Ignacio Briozzo for their support.
5. **Rosario Experimental and Behavioral Economics Lab (REBEL) at Universidad del Rosario in Bogotá.** We thank Mariana Blanco and her team for their support.
6. **Centre for Decision Research and Experimental Economics (CEDEX) at the University of Nottingham.** We thank Jose Guinot Saporta for his support.
7. **Vienna Center for Experimental Economics (VCEE) at the University of Vienna.** We thank Jean Robert Tyran and the lab's team for their support.
8. **Laboratorio de Investigación en Economía Experimental at Universidad de Valencia.** We thank Penénlope Hernández and the lab's team, including Neus Planelis, for their support.
9. **The Ohio State Experimental Economics Laboratory.** We thank John Kagel and P.J. Healy for their support.
10. **Busara Center for Behavioral Economics in Kenya.** We thank Pauline Wanjeri for her support.
11. **Experimental and Behavioural Economics Laboratory at the British University in Egypt.** We thank Dinah Rabie, Rania Miniesy, and Sarah Ahmed for their support.
12. **Shanghai Jiao Tong University.** We thank Xiangdong Qin for his support.
13. **Behavioural Business Lab at RMIT Australia.** We thank Qin Dong for her support.
14. **Cognition and Behavior Lab at Aarhus University.** We thank Lasse Lui Frandsen for his support.

References

[1] Greiner, B. Subject pool recruitment procedures: organizing experiments with orsee. *Journal of the Economic Science Association* **1**, 114–125 (2015).

[2] Bock, O., Baetge, I. & Nicklisch, A. hroot: Hamburg registration and organization online tool. *European Economic Review* **71**, 117–120 (2014).

[3] Fischbacher, U. z-tree: Zurich toolbox for ready-made economic experiments. *Experimental Economics* **10**, 171–178 (2007).

[4] Chen, D. L., Schonger, M. & Wickens, C. otree—an open-source platform for laboratory, online, and field experiments. *Journal of Behavioral and Experimental Finance* **9**, 88–97 (2016).

[5] The World Bank. World development indicators (2021). Data retrieved from World Development Indicators, `https://doi.org/10.57966/6rwy-0b07`.

[6] Baron, D. P. & Ferejohn, J. A. Bargaining in legislatures. *American Political Science Review* **83**, 1181–1206 (1989).

[7] Eraslan, H. & Merlo, A. Majority rule in a stochastic model of bargaining. *Journal of Economic Theory* **103**, 31–48 (2002).

[8] Eraslan, H. & Evdokimov, K. S. Legislative and multilateral bargaining. *Annual Review of Economics* **11**, 443–472 (2019).

[9] Herings, P. J. J., Meshalkin, A. & Predtetchinski, A. Subgame perfect equilibria in majoritarian bargaining. *Journal of Mathematical Economics* **76**, 101–112 (2018).

[10] Sen, A. *On economic inequality* (Oxford university press, 1997).

[11] Garcia-Rada, X. & Norton, M. I. Putting within-country political differences in (global) perspective. *Plos one* **15**, e0231794 (2020).

[12] Anderson, M. L. Multiple inference and gender differences in the effects of early intervention: A reevaluation of the abecedarian, perry preschool, and early training projects. *Journal of the American Statistical Association* **103**, 1481–1495 (2008).

[13] Hofstede, G. *Culture's consequences: Comparing values, behaviors, institutions and organizations across nations* (Sage, 2001).

[14] Schwartz, S. A theory of cultural value orientations: Explication and applications. *Comparative sociology* **5**, 137–182 (2006).

[15] EVS/WVS. European values study and world values survey: Joint evs/wvs 2017-2021 dataset (joint EVS/WVS) (2021).

[16] Allison, L., Wang, C. & Kaminsky, J. Religiosity, neutrality, fairness, skepticism, and societal tranquility: A data science analysis of the world values survey. *Plos one* **16**, e0245231 (2021).

[17] Beugelsdijk, S. & Welzel, C. Dimensions and dynamics of national culture: Synthesizing hofstede with inglehart. *Journal of cross-cultural psychology* **49**, 1469–1505 (2018).

[18] Kaasa, A. Merging hofstede, schwartz, and inglehart into a single system. *Journal of cross-cultural psychology* **52**, 339–353 (2021).

[19] Muthukrishna, M. *et al.* Beyond western, educated, industrial, rich, and democratic (WEIRD) psychology: Measuring and mapping scales of cultural and psychological distance. *Psychological science* **31**, 678–701 (2020).

[20] Hofstede, G., Hofstede, G. & Minkov, M. *Cultures and Organizations: Software of the Mind, Third Edition* (McGraw-Hill Education, 2010). URL `http://books.google.de/books?id=o4OqTgV3V00C`.

[21] Inglehart, R. & Baker, W. E. Modernization, cultural change, and the persistence of traditional values. *American sociological review* **65**, 19–51 (2000).

[22] Apfeld, B., Coman, E., Gerring, J. & Jessee, S. *World Values Survey/European Values Survey Questionnaire*, 285–289. Cambridge Studies in the Comparative Politics of Education (Cambridge University Press, 2024).

[23] Kirsch, H. & Welzel, C. Democracy misunderstood: Authoritarian notions of democracy around the globe. *Social Forces* **98**, 59–92 (2019).

[24] Awad, E. *et al.* The moral machine experiment. *Nature* **563**, 59–64 (2018).

[25] Schulz, J. F., Bahrami-Rad, D., Beauchamp, J. P. & Henrich, J. The church, intensive kinship, and global psychological variation. *Science* **366**, eaau5141 (2019).

[26] Kenworthy, L. & McCall, L. Inequality, public opinion and redistribution. *Socio-Economic Review* **6**, 35–68 (2008).

[27] Engerman, S. L. & Sokoloff, K. L. Colonialism, Inequality, and Long-Run Paths of Development. In *Understanding Poverty* (Oxford University Press, 2006). URL `https://doi.org/10.1093/0195305191.003.0003`. `https://academic.oup.com/book/0/chapter/272745028/chapter-ag-pdf/44536751/book_32736_section_272745028.ag.pdf`.

[28] Blekesaune, M. Economic strain and public support for redistribution: A comparative analysis of 28 european countries. *Journal of Social Policy* **42**, 57–72 (2013).

[29] Lupu, N. & Pontusson, J. The structure of inequality and the politics of redistribution. *American Political Science Review* **105**, 316–336 (2011).

[30] Rai, D., Zitko, P., Jones, K., Lynch, J. & Araya, R. Country-and individual-level socioeconomic determinants of depression: multilevel cross-national comparison. *The British Journal of Psychiatry* **202**, 195–203 (2013).

[31] Abadie, A. Poverty, political freedom, and the roots of terrorism. *American Economic Review* **96**, 50–56 (2006).

[32] Fréchette, G., Kagel, J. H. & Morelli, M. Behavioral identification in coalitional bargaining: An experimental analysis of demand bargaining and alternating offers. *Econometrica* **73**, 1893–1937 (2005).

[33] Drouvelis, M., Montero, M. & Sefton, M. Gaining power through enlargement: Strategic foundations and experimental evidence. *Games and Economic Behavior* **69**, 274–292 (2010).

[34] Kagel, J. H., Sung, H. & Winter, E. Veto power in committees: an experimental study. *Experimental Economics* **13**, 167–188 (2010).

[35] Miller, L., Montero, M. & Vanberg, C. Legislative bargaining with heterogeneous disagreement values: Theory and experiments. *Games and Economic Behavior* **107**, 60–92 (2018).

[36] Fréchette, G., Kagel, J. H. & Morelli, M. Nominal bargaining power, selection protocol, and discounting in legislative bargaining. *Journal of Public Economics* **89**, 1497–1517 (2005).

[37] Miller, L. & Vanberg, C. Decision costs in legislative bargaining: an experimental analysis. *Public Choice* **155**, 373–394 (2013).

[38] Baranski, A. & Kagel, J. H. Communication in legislative bargaining. *Journal of the Economic Science Association* **1**, 59–71 (2015).

[39] Bradfield, A. J. & Kagel, J. H. Legislative bargaining with teams. *Games and Economic Behavior* **93**, 117–127 (2015).

[40] Miller, L. & Vanberg, C. Group size and decision rules in legislative bargaining. *European Journal of Political Economy* **37**, 288–302 (2015).

[41] Baranski, A. & Haas, N. The timing of communication and retaliation in bargaining: An experimental study. *Journal of Economic Psychology* **96**, 102621 (2023).

[42] Fréchette, G. R., Kagel, J. H. & Lehrer, S. F. Bargaining in legislatures: An experimental investigation of open versus closed amendment rules. *American Political Science Review* **97**, 221–232 (2003).

[43] Agranov, M. & Tergiman, C. Communication in multilateral bargaining. *Journal of Public Economics* **118**, 75–85 (2014).

[44] Baranski, A. & Morton, R. The determinants of multilateral bargaining: A comprehensive analysis of Baron and Ferejohn majoritarian bargaining experiments. *Experimental Economics* **25**, 1079–1108 (2022).
